# Fine-scale metabolic discontinuity in a stratified prokaryote microbiome of a Red Sea deep halocline

**DOI:** 10.1038/s41396-021-00931-z

**Published:** 2021-03-01

**Authors:** Grégoire Michoud, David Kamanda Ngugi, Alan Barozzi, Giuseppe Merlino, Maria Ll. Calleja, Antonio Delgado-Huertas, Xosé Anxelu G. Morán, Daniele Daffonchio

**Affiliations:** 1 Red Sea Research Center, King Abdullah University of Science and Technology, Thuwal, Saudi Arabia; 2 Leibniz Institute DSMZ – German Collection of Microorganisms and Cell Cultures, Braunschweig, Germany; 3 Department of Climate Geochemistry, Max Planck Institute for Chemistry (MPIC), Mainz, Germany; 4 Instituto Andaluz de Ciencias de la Tierra, CSIC-UGR, Armilla, Spain; 5 Instituto Español de Oceanografía (IEO), Centro Oceanográfico de Gijón/Xixón, Gijón/Xixón, Spain

**Keywords:** Microbial ecology, Metagenomics

## Abstract

Deep-sea hypersaline anoxic basins are polyextreme environments in the ocean’s interior characterized by the high density of brines that prevents mixing with the overlaying seawater, generating sharp chemoclines and redoxclines up to tens of meters thick that host a high concentration of microbial communities. Yet, a fundamental understanding of how such pycnoclines shape microbial life and the associated biogeochemical processes at a fine scale, remains elusive. Here, we applied high-precision sampling of the brine–seawater transition interface in the Suakin Deep, located at 2770 m in the central Red Sea, to reveal previously undocumented fine-scale community structuring and succession of metabolic groups along a salinity gradient only 1 m thick. Metagenomic profiling at a 10-cm-scale resolution highlighted spatial organization of key metabolic pathways and corresponding microbial functional units, emphasizing the prominent role and significance of salinity and oxygen in shaping their ecology. Nitrogen cycling processes are especially affected by the redoxcline with ammonia oxidation processes being taxa and layers specific, highlighting also the presence of novel microorganisms, such as novel Thaumarchaeota and anammox, adapted to the changing conditions of the chemocline. The findings render the transition zone as a critical niche for nitrogen cycling, with complementary metabolic networks, in turn underscoring the biogeochemical complexity of deep-sea brines.

## Introduction

Deep hypersaline anoxic basins (DHABs) are bodies of water in the ocean’s floor with salinities up to ten times higher than the surrounding seawater [1]. They are widespread in basins, such as the Gulf of Mexico, the Mediterranean Sea, and the Red Sea [2]. The brine density, ranging from ~1 to 1.35 kg m^–3^, prevents mixing with the overlying seawater [3], forming the brine–seawater interface (BSI), which can span from less than one to tens of meters. BSI has sharp gradients of salinity, dissolved oxygen (DO) concentration and different combinations of electron donors and acceptors, and represents the microbially active and productive layer of DHABs [4].

The biogeochemical activities of prokaryotes localized in distinct DHABs reveal a complexity of microbial-driven processes, such as, but not limited to, acetogenesis, methanogenesis, denitrification, and sulfate reduction, particularly inside the BSI [4–11]. Among these metabolic processes, the presence of various nitrogen compounds belonging to all the redox states supports the hypothesis that numerous reactions of the nitrogen cycle occur in the BSI [8, 10, 12–16]. Indeed, evidence based on analyses of bulk samples demonstrated the activity of microorganisms and the presence of key enzymes recycling inorganic nitrogen in DHABs [9]. In addition, the predominance of discrete genotypes of ammonia-oxidizing archaea (AOA) in the BSI suggests that salinity is a key factor in the niche speciation of marine AOA [12, 17]. Denitrifiers are also abundant in DHABs and significantly contribute to N_2_ gas production as determined by isotopic measurements [10]. The assimilation of ammonia through several pathways was also suggested to be key for production of nitrogen-containing osmolytes, which are vital for coping with such high salinities [18].

However, while these pioneering studies have provided insights into the biogeochemical processes and key microbial communities along the BSI, many were not tailored to robustly address the fine-scale assembly and functions of microbial communities along the BSI due to lack of appropriate sampling procedures [4, 6, 7, 19], disregarding the role of steep oxic–anoxic boundaries in shaping microbial life and structure–function relationships [20] and the significance of salinity extremes in restricting metabolic diversity [21].

Here, we employed metagenomics to investigate the metabolic potential of microbial communities present in the BSI of the Suakin Deep as a model DHAB, for which only geochemical data exists since its discovery in the central Red Sea in 1969 [22–24]. High-precision sampling coupled with high-throughput sequencing techniques was used for the first time at a 10-cm-scale resolution, to disentangle prevailing microbial taxa and their functional traits along the halocline, with a focus on metabolic pathways of the nitrogen cycle.

## Materials and methods

### Sampling

Brine water samples were collected from Suakin Deep (19.61 N, 38.73 E) with the KAUST R/V Thuwal in April 2016 and 2017. Samples were collected using a Niskin Rosette sampler equipped with an Idronaut® CTD calibrated for brine water and 23 Niskin bottles, similarly as Daffonchio et al. [4]. Briefly, a first cast was performed to identify the BSI along the seawater column looking to the sharp increase of salinity that occurs between the transition from seawater and brine water. During this cast, 10 l of seawater overlaying the brine (50 m above) and 5 l of the brine water (at least 20 m below the BSI) were sampled in triplicate. On board, the water was filtered via 0.2-μm sterile polyethersulfone (PES) Sterivex^TM^ filters (Millipore Corporation) by peristaltic pumps (Millipore). A second cast was performed in order to sample and dissect the BSI by closing the Niskin bottles at different salinities (depths), from the ambient overlying seawater salinity to the brine body. Due to the small thickness of the BSI (~1 m) and the length of the Niskin bottles (90 cm), it is possible to entrap nearly all the BSI in a single Niskin bottle. However, the gradient in the BSI varies from one Niskin bottle to another due to waves and transmission speeds through the cable. Thus, to ensure that the water inside each Niskin is not mixed or only composed of seawater or brine water, we measured the salinity on top and at bottom of each bottle with a refractometer as soon as the water was on deck. Three Niskins covering most of the BSI, with the widest gradient of salinity, were selected and fractioned into 1-l bottles. Each of these 27 fractions accounted for 9.2 cm of the BSI. The 1-l fractions were vacuum filtered using reusable 1-l vacuum filter holder systems (Nalgene) through 0.2 μm PES membrane filters. The filters were stored in liquid nitrogen in 2 ml tubes containing lysis buffer (EDTA 40 mmol l^−1^ pH 8.0, Tris-HCl 50 mmol l^−1^ pH 8.0, sucrose 0.75 mol l^−1^) for further analysis. The filtered water collected at the bottom of the percolators was transferred into 15 ml Falcon tubes and stored at −20 °C for chemical analysis. Filtered and unfiltered seawater was collected in pre-combusted glass vials, acidified to pH between 1 and 2, and stored at 4 °C for total and dissolved organic carbon (DOC) and total and dissolved nitrogen (TDN) analysis in the laboratory, as in Calleja et al. [25]. Filtered and unfiltered results were not significantly different indicating that >95% of all C and N was in dissolved phase. In parallel, triplicate seawater and brine samples of 1.62 ml per each depth were fixed with 180 μl of paraformaldehyde and glutaraldehyde fixing solution for a total volume of 1.8 ml per sample, and stored in liquid nitrogen for enumeration of microbial cells using flow cytometry, as in Duarte et al. [26]. The gating strategy is shown in Supplementary Fig. 1. The differences between the salinity of the BSI in April 2016 and April 2017, and between the salinity of the fractions and the CTD data were tested using a nonparametric generalized additive model with restricted maximum likelihood (GAM, R package “mgcv” [27]).

### Geochemical analysis

Temperature, salinity, pH, concentration of DO, and hydrostatic pressure of the sampled brine pools were directly measured by the Idronaut**®** CTD mounted on the Rosette sampler. The salinity and pH of the 1-l fractions were determined using a portable multiparameter detector (YSI). Analysis of the nutrient concentrations along the BSI was enabled through the commercial service provided by GEOMAR at the Helmholtz Centre for Ocean Research Kiel (Germany).

### Stable isotopes

Isotopic measurements were carried out at the Biogeochemistry Stable Isotope Laboratory of Andalusian Earth Sciences Institute (CSIC). For total inorganic carbon (TIC) an aliquot of sample was injected into 12-ml vials prefilled with helium and five drops of 65% phosphoric acid, and shaken in a vortex agitator for 30 s. The vials were left at room temperature between 15 and 36 h to obtain a state of equilibrium [28]. The CO_2_ was separated from other residual gases by chromatography using a helium carrier gas in a Gas Bench (Thermo-Finnigan, Bremen, Germany) system interfaced with a mass spectrometer Delta XP isotope ratio mass spectrometer (Thermo-Finnigan, Bremen). Analytical precisions were estimated to be within 0.1‰ for TIC. For nitrates, water was filtered with a 0.45 μm (cellulose acetate membrane), poisoned with mercuric chloride, and stored in vials of 12 ml. Nitrogen and oxygen isotopic compositions of nitrate were analyzed by the denitrification method [29]. International reference materials (IAEA-N3, USGS-34, and USGS-35) and five in-house working standards were included within a batch of 96 bottles for a series of analysis. The N_2_O produced was then analyzed using a Thermo-Finnigan Delta V Plus. Analytical precisions were estimated to be within 0.3‰ for δ^15^N and δ^18^O.

### DNA extraction

Total DNA was extracted from the different filters using phenol–chloroform protocol [30]. Briefly, 20 mg of lysozyme were added to the filter and incubated at 37 °C in a water bath for 30 min. After this step, 20 mg of proteinase K and a final 20% SDS solution were added, and then incubated at 55 °C for 2 h in a water bath. The supernatant was transferred to a new sterile 15 ml falcon tube and one volume of phenol:chloroform:isoamyl alcohol (25:24:1, pH 7.7–8.3) was added. The sample was vortexed for 30 s and centrifuged at 8000 × *g* for 10 min. The aqueous phase was then transferred to a 15 ml falcon tube followed by a second phenol:chloroform:isoamyl alcohol extraction step. One volume of chloroform:isoamyl alcohol (24:1) was added to the resultant aqueous phase followed by vortexing and centrifugation for 10 min. The DNA in the aqueous phase was precipitated overnight using two volumes of ice-cold 100% ethanol and 1/10 volume of sodium acetate (3.0 mol l^−1^ pH 5.3) at −20 °C. The DNA was centrifuged at 4 °C for 30 min, and then washed twice with ice-cold 80% ethanol and centrifuged at 4 °C before resuspension in sterile 100 μl Milli-Q water. The concentration of DNA in each sample was quantified with Qubit® 3.0 Fluorometer using the Qubit® dsDNA BR assay kits (Thermo-Fisher Scientific), while the quality was assessed by gel electrophoresis with 1% agarose. Finally, the extracted DNA was stored at −20 °C until further analyses. The amount of DNA per filter was between 70 and 1540 ng.

### Metagenomic library preparation, sequencing, and analyses

Metagenomic libraries were prepared with the Ovation Ultralow V2 DNA-Seq Library preparation kit (NuGen), following the manufacturer’s instruction. First, between 50 and 100 ng of gDNA were diluted in 120 μl of nuclease-free water (Ambion), in Covaris snap cap microtube (PN 520045, Covaris). The gDNA was fragmented by sonication with Covaris M220 to target DNA fragments of 300 bp, following this sonication protocol: treatment time 70 s, cycle per bust 200, duty factor 20%, peak incident power 50 W. The 300 bp fragmented DNA was used as impute to prepare metagenomic libraries. The quality of each library was checked with Bioanalyzer 2100 (Agilent) and the amount of DNA quantified with Qubit 3.0 (Thermofischer). The prepared libraries were pooled together in a sample pool with a final concentration of 20 nmol l^−1^.

The quality and quantity of the library pool were checked with Bioanalyzer 2100 (Agilent) and qPCR with the Kapa Library Quantification kit (KAPA Biosystems) respectively. The sequencing was performed with the Illumina Hiseq 4000 platform (2 × 150 bp cycle) at the Biological Core Lab at King Abdullah University for Science and Technology (KAUST). Raw read sequences were quality filtered and trimmed using Trimmomatic v0.32 [31] to remove adapter sequences and leading and trailing bases with a quality score <20 and reads with an average per base quality of 20 over a 4-base pair (bp) window. This preprocessing step also included a mapping-based step to remove reads from the internal phage standard PhiX, using BBmap v37.44 [32] with default settings. At each step, sequence quality was assessed with FASTQC v0.11.7 [33]. To determine microbiome diversity and community structure, SingleM v0.9.0 was applied to reads from each sample (Woodcroft B.J. et al., unpublished materials, source code available at https://github.com/wwood/singlem). De novo OTUs were generated using 14 single-copy genes (SCGs) and clustering of mapped reads at 89% identity (genus level), to avoid sequencing errors. The assigned taxonomy is based on the nomenclature implemented in the GDTB database [34]. Beta diversity based on the normalized abundance of the 14 SCGs was then assessed by weighted Unifrac beta diversity with the Express Beta Diversity software v1.0.8 [35]. The PcoA was made in R with the ape package [36].

The resulting high-quality reads for each dataset were independently assembled using metaSPAdes v3.9.0 [37], employing the error-correction mode and preset metagenomic options, using a kmer range of 21–127. The assembled contigs were then filtered to a minimum length of 500 bp prior to gene prediction using Prodigal v2.6.2 [38], with the options “-p meta -m”. The predicted-coding genes for all 11 individual samples totaled 346,740 genes, averaging (±SD) 32,304 ± 8923 genes per sample for genes with a minimum length of 100 bp (Supplementary Table 1).

### Quantification of gene family abundance across the salinity gradient

The predicted genes (≥100 bp) from all samples were concatenated into a single fasta file and dereplicated using cd-hit-est [39] by applying a global identity cutoff of 95% and a coverage of 80% for the shorter gene. This yielded a catalog of 117,573 nonredundant genes, which we refer to as the “Suakin Microbial Gene Catalog” (SMGC). The error-corrected reads from each sample were subsequently mapped against this gene catalog with bowtie2 v2.3.3 [40], applying the “--sensitive” and “--qc-filter” parameters in addition to the default settings to generate a table of reads counts per gene across the sampled salinity gradient. The reads-per-gene count table was then normalized using the average genome size in each sample, and the respective gene lengths yielding a common metric of reads per kilobase per genome equivalent (RPKG) across all metagenomes, as described by Nayfach and Pollard [41]. The average genome sizes were estimated using the kmer frequency in 10 million randomly sampled paired-end error-corrected reads in each metagenomes, using MicrobeCensus v1.0.7 [41].

To evaluate the stratification in microbial assembly and metabolic functions, we annotated the gene catalog using DMAP (http://www.cbrc.kaust.edu.sa/dmap/index.php; [42]), applying a blast score cutoff value of 70 to assign genes to functions based on the KEGG orthology (KO) database and probable taxonomic origin based on the lowest-common-ancestor approach. The RPKG (normalized abundance) data was then integrated with the assigned KEGG functions and taxa for each gene to profile the distribution individual pathways in the sample metagenomes across the salinity gradient.

In-depth functional analyses focused primarily on three biogeochemical cycles involving carbon (C), nitrogen (N) and sulfur (S) metabolism. Briefly, the genetic potential of the resident microbial community was analyzed by based on abundance and taxonomic assignment of marker genes (or KOs) encoding key enzymes various pathways, such as anaerobic ammonia oxidation (Anammox), nitrification, methane oxidation, and sulfate reduction among others (Supplementary Table 2) [43]. Most of the enzymes used can be separated using KOs, with the exception of the nitrate reductases and nitrite oxidoreductases (*narG*/*nxrA*; K00370). We thus followed the procedure of Lüke et al. to distinguish the two genes [44]—that is, genes that were assigned to the KO K00370 were blasted against two *nxrA* gene subsets, one containing the *Nitrobacter*/*Nitrococcus*/*Nitrolancea* sequences and the other containing the *Nitrospira*/*Nitrospina*/anammox sequences. A blast score ratio of 0.85 was then used to separate *nxrA* gene sequences of nitrite oxidizers (*Nitrobacter*/*Nitrococcus*/*Nitrolancea* and *Nitrospira*/*Nitrospina*/Anammox) from *narG* gene sequences of nitrate reducers [44]. Then, the normalized abundances of the various enzymes of a given pathway were mean-centered to aid visualization of different broad to account for pathways with very different RPKG values ranging from 0 to 32. Hierarchical clustering with the Euclidean distance metric and average linkage was used to cluster pathways with similar abundance profiles based on the normalized RPKGs. The heatmaps were made in R with the ggplot2 package [45] and modified in Inkscape.

To determine functional changes with abiotic factors and the gene catalog, we performed a canonical correspondence analysis (CCA). First, to avoid multicollinearity of covarying factors, we used a spearman correlation between all of them (Supplementary Table 3). Consequently, the colinear variables were removed from downstream analyses. The vegan package [46] in R was used for stepwise variable selection based on the AIC criterion based on the method of Blanchet et al. [47], which allowed the identification of physicochemical parameters contributing significantly to the observed community variance and the CCA analysis.

### Recovery of population genomes

Metagenome-assembled genomes (MAGs) were recovered from the assembled contigs of each sample with Metabat2 v2.12.1 [48] based on reads coverage information obtained by mapping the error-corrected reads of individual samples to the respective contigs, using bowtie2 with the above settings. The resulting MAGs were subsequently refined, using RefineM v0.0.22 (https://github.com/dparks1134/RefineM). Briefly, for each MAG, contigs with divergent genomic properties (GC%), tetranucleotide and coverage were removed, then those remaining were taxonomically classified against a reference database using DIAMOND v0.9.14 [49], and removed if they were taxonomically diverging from MAG. The recovered MAGs that were taxonomically highly identical in the sampled gradient were subsequently dereplicated with dRep v2.3.2 [50], with the “dRep dereplicate” command. This command uses the completeness and contamination estimate from the CheckM software (v1.0.18) [51] to choose the best dereplicated MAGs. Those were then used to determine the relative abundance (coverage) of community members with BamM v1.7.3 (http://ecogenomics.github.io/BamM/) following the pipeline described in Woodcrof et al. [52]. Briefly, reads from each sample were mapped to the set of dereplicated genomes using BamM “make”, followed by removal of low-quality mappings using BamM “filter” (minimum identity 95%, minimum aligned length 75% of each read), and determination of contig coverage with BamM “parse” using “tpmean” mode. The coverage of each MAG was calculated as the average of contig coverages.

The dereplicated MAGs were also annotated with prokka v1.13 [53]. Taxonomic assignment of the different MAGs was conducted using the GTDB-Tk software v0.3.1, which uses 120 and 122 concatenated proteins for respectively bacteria and archaea, and the pplacer software (v1.1.alpha19) to assign genomes to taxa in the GTDB database tree [34, 54, 55] (Supplementary Table 4).

### Comparative genomics and phylogenomics

The phylogenomic trees were constructed using a concatenated set of 120 bacterial genes or 122 archaeal single-copy marker genes implemented in the GTDB-Tk software (v0.3.1) [55] based on the “classify_wf” workflow. The resulting multiple sequence alignments containing 5035 (Anammox) and 5024 (AOAs) aligned positions was trimmed, using GBlocks v0.91b [56] based on the liberal settings. The final alignments containing 851 (Anammox) and 3982 (AOAs) aligned positions were used for phylogenetic reconstruction. The best substitution model for tree construction was selected using ProTest3 [57]. A maximum-likelihood phylogenetic tree was constructed from the liberal superalignment using RaxML v7.2.8 [58] as implemented in GeneiousPro v8.1.9 (http://www.geneious.com) under the GAMMA + WAG amino acid substitution models. Tree topology was tested for robustness using 100 bootstrap replicates. The tree was visualized in FigTree [59] and modified for publication in Adobe Illustrator. The trees were rooted using *Rhodopirellula baltica* SH 1 (accession number BXX119912) and *Pyrococcus furiosus* DSM 3638 (AE009950) for the anammox and the thaumarchaeal trees, respectively.

The average amino acid identity (AAI) between reconstructed MAGs an in relation to reference genomes were calculated using the phylogenomic pipeline “Get_homologues” v20092018 [60] based on orthologues generated, using the OrthoMCL v1.4 software [61]. The AAI matrix was then imported into the R software environment and plotted using heatmap.2 package from the library gplots [62]. The isoelectric point (pI) of predicted proteomes, in particular, the putative cytoplasmatic protein-coding genes was computed, as described in Ngugi et al. [13].

### Quantitative PCR analysis

The quantification of nitrogen cycle-related functional genes, archaeal and bacterial ammonia monooxygenase subunit A (*amoA*) and *hzo* was carried out following the methods described by Duarte et al. [26] and elsewhere [63, 64]. Briefly, the fragments of interest were amplified from total genomic DNA isolated from environmental samples by PCR and cloned into into pCR™2.1-TOPO® vectors. Clones were screened using the β-galactosidase assay, selecting and re-streaking white colonies. Positive clones were stored at −80 °C in a 20% final concentration glycerol solution.

DNA plasmids were isolated from overnight-grown clone cultures using Pure Yield Plasmid Miniprep (Promega). The isolated vectors were quantified using the Qubit dsDNA HS Assay Kit (Thermo-Fisher Scientific). The obtained concentrations (in ng μl^−1^) were used to calculate the concentration of the plasmids in terms of copies μl^−1^ and to prepare the series of standards used in the qPCR runs.

Quantitative PCR reactions were setup with a robotic Qiagility workstation (Qiagen) and were carried out on a Rotor-Gene Q thermocycler (Qiagen). All the samples were first quantified with Qubit dsDNA BR Assay Kit. Dilutions to 0.5 ng μl^−1^ of each sample were prepared as template DNA for the qPCR runs. Reaction mix were prepared with the GoTaq® qPCR Sybr Green Master Mix (Promega) as indicated by the manufacturer’s instructions. Quantitative PCR conditions were the following: 95 °C for 2 min, 45 cycles at 95 °C for 15 s, annealing at the primer set-specific annealing temperature for 20 s and 60 °C for 20–45 s (depending on the length of the target fragment); after the end of the run, denaturation curves were obtained performing renaturation at 50 °C for 180 s, followed by gradual denaturation for 91 cycles from 50 °C to 95 °C with increase of 0.5 °C per cycle every 5 s.

A standard curve was constructed in every qPCR assay with a series of dilutions raging from 5 × 10^1^ to 5 × 10^7^ copies μl^−1^. All the standards and samples were run in triplicates. Concentration in terms of copies of gene μl^−1^, for every sample was calculated from the normalized standard curve. In all the qPCR runs, amplification efficiencies varied between 0.87 and 1.03, and R2 varied between 0.993 and 0.9997.

### Data deposition

The draft genome sequences have been deposited in GenBank under accession numbers JAANWX000000000-JAANXI000000000, while the metagenomic datasets are under the BioProject number PRJNA593704. The corresponding raw sequences are present in the Short Reads Archives under accession number SRR10589498-508. Flow cytometry data are available in.fcs format online (http://flowrepository.org) under the “FR-FCM-Z2LZ”.

## Results and discussion

### Environmental parameters

**Fig. 1:**
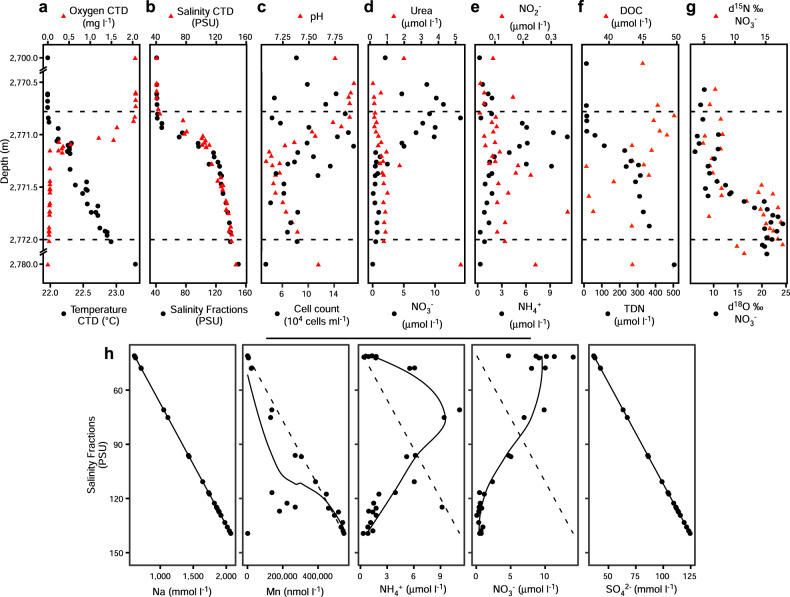
Vertical profiles of physicochemical conditions and abundance of prokaryotes in the overlaying water, along the BSI, and in the brine water. Additional data provided in Supplementary Table 1. **a** Black circles, temperature; red triangles, oxygen concentration. **b** Black circles, salinity obtained from the fractions; red triangles, Salinity obtained from an independent CTD cast. **c** Black circles, prokaryotic cell count; red triangles, pH. **d** Black circles, nitrate concentration; red triangles, urea concentration. **e** Black circles, ammonia concentration; red triangles, nitrite concentration. **f** Black circles, TDN concentration; red triangles, DOC concentration. **g** Black circles, isotopic composition of oxygen (δ^18^O) in nitrate; red triangles, isotopic composition of nitrogen in nitrate. The horizontal dash line represents the limits of the BSI. **h** Relationship between sodium, manganese, ammonia, nitrate, and sulfate in function of salinity. The oblique dash line represents the correlation between sodium and salinity for visualization purpose.

The brine of the Suakin Deep, the deepest point of the Red Sea [65], is located at a depth of 2771 m below sea level in the central Red Sea (19.61 N, 38.73 E). Steep, opposite gradients of salinity, temperature, and DO characterize the BSI spanning only 1 m in depth (Supplementary Fig. 2). As expected from past sampling and literature data [22, 24], salinity in the brine body is 150 practical salinity units (PSU), 3.5 times higher than in the overlying Red Sea water at this depth (42 PSU). In spite of the steep salinity gradient, temperature increased only marginally (from 22.0 °C to 23.3 °C), whereas DO was below the detection limit (0.01 mg l^−1^) in the brine body (Fig. 1a and Supplementary Fig. 2). Results from two sampling campaigns performed a year apart (in 2016 and 2017) confirmed that the basin of Suakin is very stable in terms of salinity gradient over time (generalized additive model, GAM; *F* = 2.3, *P* = 0.54, Supplementary Fig. 3). Thus, hereafter, we focused the analysis on the data obtained in April 2016 due to the large amount of data obtained during this campaign with the exception of the stable isotope measures, which were obtained in 2017.

The BSI was sampled using Niskin bottles coupled with a CTD probe, which were closed at different depths tailored at entrapping all of the BSI layers in different Niskin bottles (see “Methods” section). Comparing the salinity of fractionated BSI gradient samples with the CTD data (Fig. 1b) showed that these two approaches yielded virtually identical salinity profiles (GAM; *F* = 0.18, *P* = 0.67), effectively confirming the highly stratified interface layer and the stability of the fractions sampled from each independent Niskin bottle. The staggered sampling was able to capture nearly 90% of the salinity gradient within the BSI of 40–140 PSU. Figure 1 and Supplementary Table 5 summarize the geochemical properties of samples taken along the BSI. As described previously [1, 24] and confirmed here, the Suakin brine is thalassohaline as its major dissolved ions are those of the seawater (sodium and chloride). Furthermore, the stratification of the fractions was further confirmed by a very high correlation (> 0.99) between major ions, such as sodium, chloride, magnesium, and calcium and salinity (Supplementary Table 3). The pH was relatively circumneutral with a decrease up to the middle of the BSI at about seven compared to the seawater at 7.75, and then an increase at 7.5 in the brine body (Fig. 1c). The number of prokaryotic cells across the BSI ranged from 3.4 × 10^4^ to 1.7 × 10^5^ cells ml^−1^ with the highest values in the BSI and the lowest in the brine body (Fig. 1c). These values are similar to those measured in other DHABs in the Red Sea [15], but lower than the 10^6^–10^7^ cells ml^−1^ measured in the BSI of DHABs in other oceans [3, 4, 6, 7].

The concentration of urea was twofold higher in the brine body (5 μmol l^−1^) than in the overlaying seawater (2 μmol l^−1^; Fig. 1d), and remained mostly <1 μmol l^−1^ inside the BSI. The high urea concentration in the brine body contrasts with other Red Sea brines (e.g., the Discovery Deep and Erba Deep), where it reached only 1.5–1.7 μmol l^−1^ [12]. Nitrate concentrations were highest in the upper oxic layers of the BSI (up to 15 μmol l^−1^), but decreased notably in the lower anoxic layers (<2.4 μmol l^−1^); values were below the detection limit (>0.1 μmol l^−1^) in the brine body. Ammonia and nitrite were elevated in the middle (70 PSU) and in the lower part of the BSI, respectively (Fig. 1e). The increasing concentration trend of some reduced N species (i.e., urea and NO_2_^−^) in the deeper part of the BSI and especially the brine body was accompanied by a parallel increase of TDN (Fig. 1f), often observed under low oxygen concentrations in stratified waters or sediments [66, 67]. Of note, is the large increase in TDN from ~15 μmol l^−1^ in the overlaying water to values between 100 and 300 μmol l^−1^ in the BSI to higher than 400 μmol l^−1^ in the brine body. The much lower values of inorganic nitrogen species and urea (10–30 μmol l^−1^) indicate a large accumulation of as-yet undetermined abundant dissolved organic nitrogen compounds in the brine, possibly amino acids and proteinaceous material, as evidenced also in Antarctic sea ice brines [68]. On the contrary, DOC gradually decreased with depth along the BSI (from 45.9 to 36.7 μmol l^−1^), suggesting the use of dissolved carbohydrates as substrates for heterotrophic microbes within the BSI. The inverse correlation between DOC with TDN (*r* = −0.69, *P* < 0.01) suggests a tendency for ammonification at higher C concentrations as deduced in other ecosystems [69]. The positive correlation of both δ^15^N and δ^18^O of nitrate with depth (*r* > 0.70, *P* < 0.001, Fig. 1g and Supplementary Table 3) indicates the prevalence of denitrification, especially in the upper zone of the BSI and the brine body [70]. Samples below the ammonification zone are characterized by low concentrations of residual nitrates and high isotopic values of both δ^15^N and δ^18^O (Supplementary Fig. 4). These two isotopic compositional groups could be related to two processes in the formation of nitrates: (a) oxidation of nitrites, in the most oxidizing zone (samples ~1:1 line, upper BSI), and (b) anaerobic ammonium oxidation (anammox) in the most reducing zone (samples with relatively higher δ^18^O values, plot at the left of the 1:1 line).

With the exceptions of pH and nitrogen species described previously, most of the chemical species measured here tends to be more abundant in the brine body or the lower part of the BSI than in the upper part (Fig. 1 and Supplementary Table 5). This suggests that the sinking organic and inorganic particulate matters coming from the overlaying seawater are temporarily trapped in the BSI and tend to accumulate at the bottom of the BSI. The high correlation along the BSI of several of these chemical species with salinity or components of salinity, such as sodium or chloride ions, suggests that their concentrations are marginally affected by the microbiome of the BSI. Indeed, similarly to Bannock DHAB [4], Na^+^ profiles showed a chemically conservative behavior along the BSI of the Suakin Deep, which is different from manganese, ammonia, and nitrate that showed nonlinear slopes, reflecting active biological redox cycling (Fig. 1h). However, the sulfate profile showed a linear slope with salinity in contrast geochemical data from Bannock [4], suggesting limited sulfate reduction effect consistent with the absence of sulfides [24]. Some of the elements not correlating with salinity, such as iron, copper, lead, and aluminum are only present in traces or even below the detection limit (Supplementary Table 5). The case of lead is particularly interesting as it is at low concentration in the BSI (<100 pmol l^−1^), but relatively abundant in the brine body (656.37 pmol l^−1^), which suggests the presence of a lead source in the brine of the Suakin Deep reminiscent of its hydrothermal origin [24].

### Taxonomic structure of the microbial communities

**Fig. 2:**
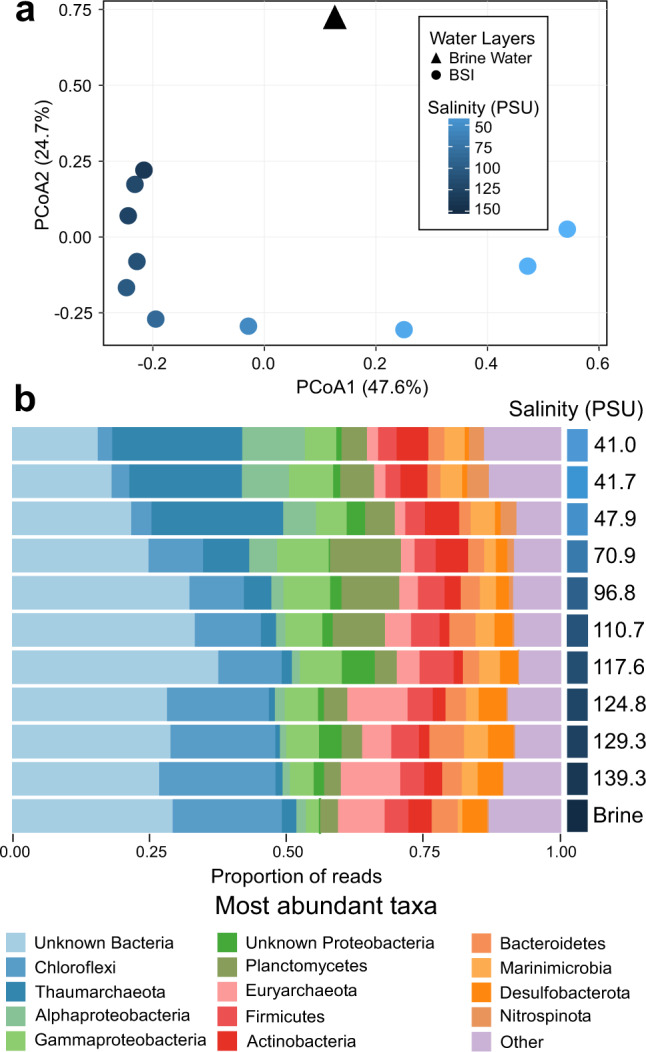
Microbial composition and diversity along the halocline. **a** Principal component analysis (PCoA) of weighted UniFrac distances comparing the different samples obtained from the BSI (circles) and brine water (triangle) based on their taxonomy at the genus level. The different samples are colored by a gradient of salinity. **b** Prokaryotic community structure (relative abundances at the phylum and classes level) of BSI. The fractions are ordered by increasing salinity.

The sampling of the 1-m-thick salinity gradient encompassed eleven distinct fractions within the BSI layer down to the brine body, allowing fine-scale community composition and functional profiling by whole-metagenome shotgun sequencing. The 11 metagenomes produced 63 Giga-base pairs (Gbp) of sequence data, with an average 4.7 Gbp per each BSI layer (Supplementary Table 1). Taxonomic analysis based on de novo operational taxonomic units (clustered at genus level, 89%) generated using 14 SCGs [35] revealed the predominance of bacteria (80%) over archaea (Fig. 2), as previously observed in other Red Sea brines [8]. The assessment of the microbial communities using the weighted Unifrac beta diversity metric based on all SCGs showed grouping of samples by their local salinity (Fig. 2a). The ordinations explained 72.3% of the total compositional variation of the samples. We noted a relatively high number of taxa that could not be assigned to a specific phylum (unknown bacteria) or class (unknown Proteobacteria) that is likely attributable to the underexplored nature of the Red Sea brines, where several novel uncultivated taxa have been discovered in several studies [1–3, 71–73].

The majority of the sequences in the microbiome with abundance higher than 10% belonged to Chloroflexi, Thaumarchaeota, Gammaproteobacteria, and Planctomycetes (Fig. 2b). Sequences affiliated with Thaumarchaeota (22.7 ± 1.9%), Alphaproteobacteria (7.8 ±2.8%), and Actinobacteria (5.7 ± 0.6%) predominated in the upper part of the BSI, reducing in abundance as the salinity increased to 80 PSU (middle BSI), whereas Chloroflexi (15.3 ± 4.8%), Firmicutes (4.9 ± 0.6%), and Euryarchaeota (5.4 ± 2.8%) increased in abundance in the lower part of the BSI and the brine body (Fig. 2b). The phylum Thaumarchaeota is mainly composed of the family Nitrosopumilaceae (93% of this taxa). This family includes aerobic ammonia oxidizers, which were shown to be present in the BSI of other Red Sea DHABs such Atlantis II and Kebrit [12]. The families Anaerolineaceae and envOPS12 in the Chloroflexi phylum are anaerobic taxa usually found in hydrothermal vents, and marine subsurface sediment [74, 75]. Interestingly, while 60.1% of the Euryarchaeota OTUs could not be classified to the order level, we noted that the most abundant taxa belong to the Methanonatronarchaeales (11.8%), the Halobacteriales (6%) and the Methanomicrobiales (5.1%). All these taxa increased in the relative abundance toward the bottom of the BSI, to become the most abundant at a salinity of ~130–140 PSU. All these taxa comprise anaerobic microorganisms able to withstand high saline conditions via different “salt-in” and “salt-out” osmoprotective mechanisms [76–78]. While, some of the child taxa are known methanogens, the low concentration of methane in the brine body (7.3 µl l^−1^) [24] compared to Kebrit Deep (~9.6 µl l^−1^) [24], a DHAB where methanogens have been documented [8], suggests their limited growth and/or activity in the brine of the Suakin Deep.

Noteworthy, the salinity range of ~70–90 PSU (middle of the BSI) at which the abundance of these taxa shifted, encompassed the oxic–anoxic boundary, where oxygen concentration was ~1 mg l^−1^ (Fig. 1a). The phylum Planctomycetes, however, followed a different trend, with highest abundance (11 ± 1.7%) in the middle of the BSI (~70–90 PSU), where ammonia concentration peaked (Fig. 1e). Seventy three percent of sequences within this phylum were assigned to the order Brocadiales, suggesting a niche for anammox bacteria [79]. Annamox bacteria are known to be present in the very specific niche of the oxygen minimum zones [80] and can be active at low levels of oxygen concentration [81]. Few other taxa, much less abundant followed this trend, such as the order Microtrichales (1.3% of the community), known to be relatively abundant in oxygen minimum zones [80] and the candidate phylum Patescibacteria (0.8%) also known to be found in environments with low oxygen concentrations, such as groundwaters [82].

Paradoxically, sequences affiliated with the proposed phylum Persephonarchaea (formerly “Candidate Division MSBL-1”) [72, 73] and Desulfobacteraceae [8], though canonically present in Red Sea and Mediterranean Sea brines [72, 83], were not detected in the Suakin Deep.

### Functional structure of the microbial communities

**Fig. 3:**
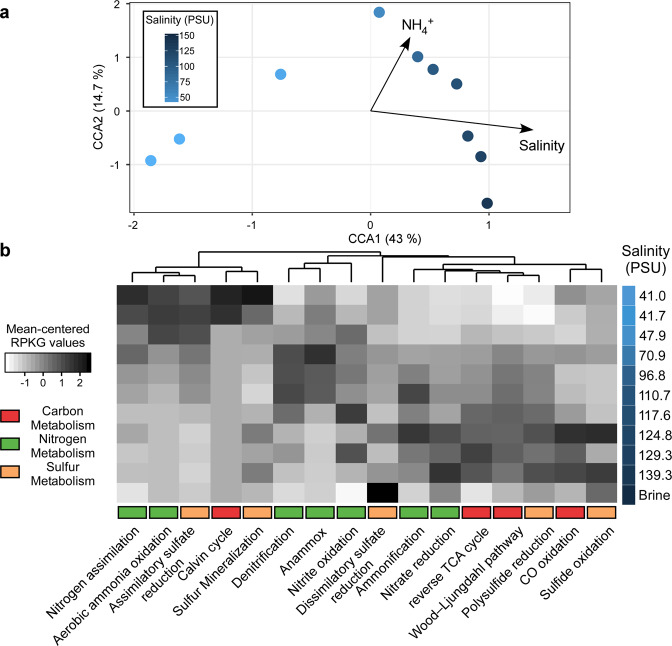
Stratification of metabolic functions along the halocline. **a** Canonical correspondence analysis (CCA) for explorative reduced models (after stepwise environmental variable selection based on the AIC criterion) comparing the different samples obtained from the BSI based on the relative abundance of the different KOs belonging to the SMGC. The different samples are colored by a gradient of salinity. **b** Heatmap of gene abundances based on selected KEGG orthologs (KOs) in the annotated gene catalog. Metabolic functions are deduced from the profiles of selected KOs presented in Supplementary Table 2. Abundances were computed using the reads per kilobase per genome (RPKG) metric. Pathway-specific RPKG values were scaled around the means for visualization purposes as the RPKG values per pathway ranged from 0 to 32. Hierarchical clustering is based on the Euclidean distance of abundances and average linkage approach. The numerous pathways are color-highlighted based on the three broad metabolic pathways: carbon (red), nitrogen (green), and sulfur (orange).

The deduced spatial stratification of microbial communities that catalyze key biogeochemical processes along the salinity gradient warrants detailed examination of their distribution and ecology. For this purpose, we generated a catalog of protein-coding genes comprised of 117,573 nonredundant genes from all 11 metagenomes, including the brine, referred to here as the SMGC. More than half of the genes (65,507 genes) in SMGC were assigned KO group labels, in turn, facilitating a functional interrogation of several metabolisms across the salinity gradient [43]. The normalized abundances of annotated marker genes (used as proxy for potential function) were correlated with measured abiotic factors and visualized using CCA. It is noteworthy that most physicochemical parameters (e.g., sulfate, strontium, sodium, chloride, calcium, magnesium, TDN, nitrate, and nitrite) significantly covaried with salinity (*p* < 0.001, Supplementary Table 3). Consequently, we selected variables significantly contributing to the observed community assembly (see “Methods” section), which showed that salinity and ammonia could respectively explain 34.9 and 17.5% of the variance (Supplementary Table 6 and Fig. 3a).

**Fig. 4:**
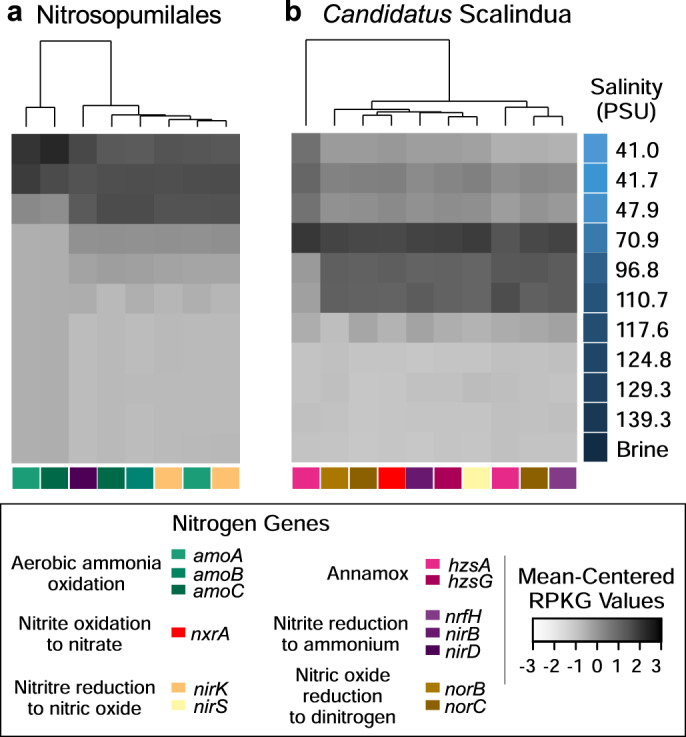
Abundance and taxonomic breakdown of nitrogen cycling enzymes along the halocline. A similar approach to the one adopted in Fig. 3 was used, but focused on genes predicted to originate from Thaumarchaea and anammox bacteria.

To elucidate metabolism stratification along the BSI, we selected several KOs as markers for key enzymes involved in carbon, nitrogen, and sulfur metabolisms [43] (Fig. 3b and Supplementary Table 2). First, genes encoding enzymes involved in aerobic methane oxidation, methanogenesis, and nitrogen fixation were absent in the gene catalog, suggesting potential lack of the corresponding metabolic groups. These results are in agreement with the low methane concentrations in the brine [24] and are consistent with lack of nitrogen fixation in oxygen minimum zones [84]. Normalized abundances of each KO based on number of mapped reads per gene scaled by the predicted average genome size of microbes in each metagenomic sample (Fig. 3 and Supplementary Table 2; details in “Methods” section) indicate the prevalence of the reverse TCA cycle, Wood–Ljungdahl, nitrate reduction, nitrite oxidation, and ammonification pathways in the anoxic part of the BSI (118–140 PSU; 0.01–0.5 mg ml^−1^ DO; Fig. 3). Other pathways observed in the lower part of the BSI were carbon monoxide oxidation, polysulfide oxidation, sulfate reduction, and sulfide oxidation. On the contrary, the nitrogen assimilation pathway along with assimilatory sulfate reduction, Calvin cycle, sulfur mineralization, and aerobic ammonia oxidation were present in the oxic part of the BSI (41–70 PSU, 2.1–1.5 mg ml^−1^ DO, Fig. 3). The genes involved in the anammox and denitrification pathways were mainly present in the middle of the interface where DO concentration ranged between 0.5 and 1.6 mg ml^−1^. The taxonomic assignment of the corresponding key enzymes along the BSI showed a clear stratification of enzymes assigned to members of the Nitrosopumilales order and the genus *Candidatus* Scalindua (Fig. 4), which respectively perform aerobic and anaerobic ammonia oxidation. The other genes putatively involved in alternative nitrogen metabolisms could not be taxonomically differentiated along the BSI.

### Genome-centric view of the ammonia metabolism

The fact that ammonia explained 17.5% of the variance of relative gene abundances across the different fractions in the BSI (Fig. 3a) and that both anammox-related metabolic genes and the phylum Planctomycetes were abundant in the middle of the BSI (Figs. 2b and 3b), led us to investigate further the fine-scale salinity-dependent effects on the microorganisms catalyzing ammonia oxidation. The independent metagenomic assemblies from the eleven samples allowed us to generate a total of 30 high-quality MAGs, six of which were affiliated with the anammox genus “*Candidatus* Scalindua”, while five were affiliated with AOA in the family Nitrosopumilaceae (Supplementary Table 4).

**Fig. 5:**
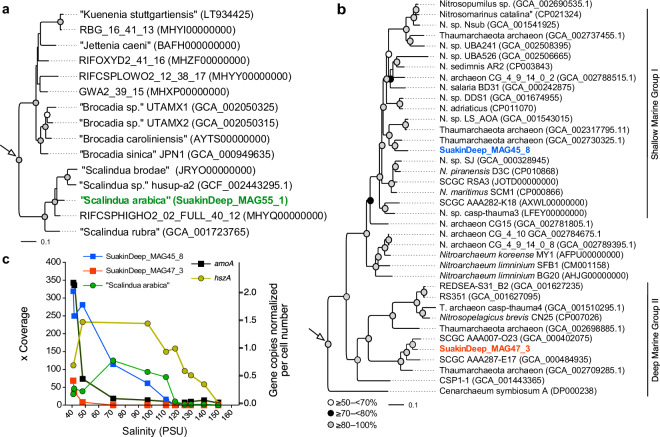
Phylogenomic placement and abundance of reconstructed genomes. Maximum-likelihood trees of **a** Anammox and **b** thaumarchaeal genomes. Reconstructed MAGs are indicated in bold. White arrows show the root in each tree using *Rhodopirellula baltica* (BXX119912) and *Pyrococcus furiosus* DSM 3638 (AE009950) as outgroups, respectively. For brevity, the “*Candidatus*” epithet is shown in parenthesis. **c** Highlights the abundance of reconstructed MAGs along the halocline based on the average genome coverage metric (mapping rate) against 11 metagenomes (40–150 PSU), and the abundance of the *amoA* and *hszA* genes copies normalized by cell number and measured by quantitative real-time PCR.

Though the MAGs encompassed independent assemblies and samples, further interrogation of orthologous genes based on the average AAI metric revealed that all the “*Ca*. Scalindua” MAGs likely originated from the same bacterial population (95–96% AAI; Supplementary Fig. 5), whereas the Nitrosopumilaceae MAGs encompassed two separate AOA lineages (Supplementary Fig. 6). Therefore, we dereplicated the “*Ca*. Scalindua” MAGs (*n* = 6) into a single genome designated as “SuakinDeep_MAG55_1” (92.9% complete; 2.27% contamination). This MAG is phylogenetically distinct from cultivated (“*Ca*. Sc. sorokinii”, “*Ca*. Sc. brodae”, and “*Ca*. Sc. wagneri” [85]) and uncultivated *Ca*. Scalindua species (“*Ca*. Scalindua rubra” and “*Ca*. Scalindua sp. AMX11” [86, 87], Fig. 5a). This led us to designate the retrieved Scalindua MAG as “*Ca*. Scalindua arabica” [85]. It is noteworthy that the expected complete genome size of “*Ca*. Scalindua arabica” averages (±SD) 3.03 ± 0.32 Mbp, implying that its full genome might be the smallest yet among the current anammox organisms (3.16–5.58 Mbp).

Similarly, the dereplication (at 95% average nucleotide identity) of thaumarchaeal MAGs yielded two distinct species belonging to the genera *Nitrosopumilus* (SuakinDeep_MAG45_8) and *Nitrosopelagicus* (SuakinDeep_MAG47_3) with genome sizes of 1.46 Mbp (94.2% complete; ~1% contamination) and 1.05 Mbp (78.4% complete; ~2% contamination), respectively. Phylogenomic analyses corroborated the discrete taxonomic assignment of both thaumarchaeal MAGs (Fig. 5b); the *Nitrosopelagicus*-like MAG has an AAI of ~94% against a single-cell genome SCGC AAA007-O23 from the Red Sea brines affiliated to the Deep Marine Group 1 lineage, whereas the *Nitrosopumilus* genome formed a sub-lineage within the Shallow Marine Group 1 (SMG1) lineages. The distant phylogenetic relationship of this species with previous genomes recovered from brine samples in the Red Sea (e.g., SCGC RSA3) [12] combined with the lower AAI values of orthologous genes (78–80%), implies shifts in SMG1 genotypes occupying geochemically distinct DHABs.

The abundance of all three MAGs based on the mapping rate (coverage) against metagenomic sequences (95% nucleotide identity and 75% alignment coverage) revealed that the “*Nitrosopelagicus*” species were constrained to the upper part of the BSI (up to a salinity of ~50 PSU), while the “*Nitrosopumilus*” species were highly represented (coverage 50×) up to a salinity of 97 PSU (Fig. 5c). In the shallower part of the BSI (salinity: 40–47 PSU), where “*Nitrosopelagicus*” species had a high coverage (8–69×; Fig. 5c), we observed that the coverage for the “*Nitrosopumilus*” species was 4- to 35-fold higher, suggesting a larger population size relative to the former species. Interestingly, we observed an inverse relationship of genome coverage and salinity for both “*Nitrosopumilus*” and “*Nitrosopelagicus*” MAG (*r* = −0.95 and *r* = –0.79, *P* < 0.004), but not for the “*Ca*. Scalindua arabica” MAG (*r* = –0.43; *P* = 0.19). The mapping coverage showed that the “*Ca*. Scalindua arabica” genome was highly represented along the halocline (up to 118 PSU; Fig. 5c), with a peak ~70–110 PSU (mapping rate of 8–69×; Fig. 5c), implying a lifestyle adapted to a broader salinity range compared to the thaumarcheal counterparts. Complementary results based on quantitative PCR-derived copy numbers of genes encoding for *amoA* and the hydrazine synthase subunit A (*hszA*)—the marker genes for aerobic ammonia oxidation (performed by “*Nitrosopumilus*” sp.) and anammox (performed by “*Ca*. Scalindua arabica”), respectively, confirmed the taxa stratification based on metagenomic read sequence coverage (Fig. 5c).

A positive correlation was also found between the concentration of dissolved nitrate (*R* = 0.53; *P* = 0.011) and average genome coverage for “*Nitrosopumilus*” MAGs along the halocline, suggesting that this AOA species is potentially a major driver of ammonia and nitrate fluxes. The distribution of this AOA species in the high-salinity zones of the BSI by AOA species is strongly affected by the increasingly reducing conditions, in line with recent proposals [88].

**Fig. 6:**
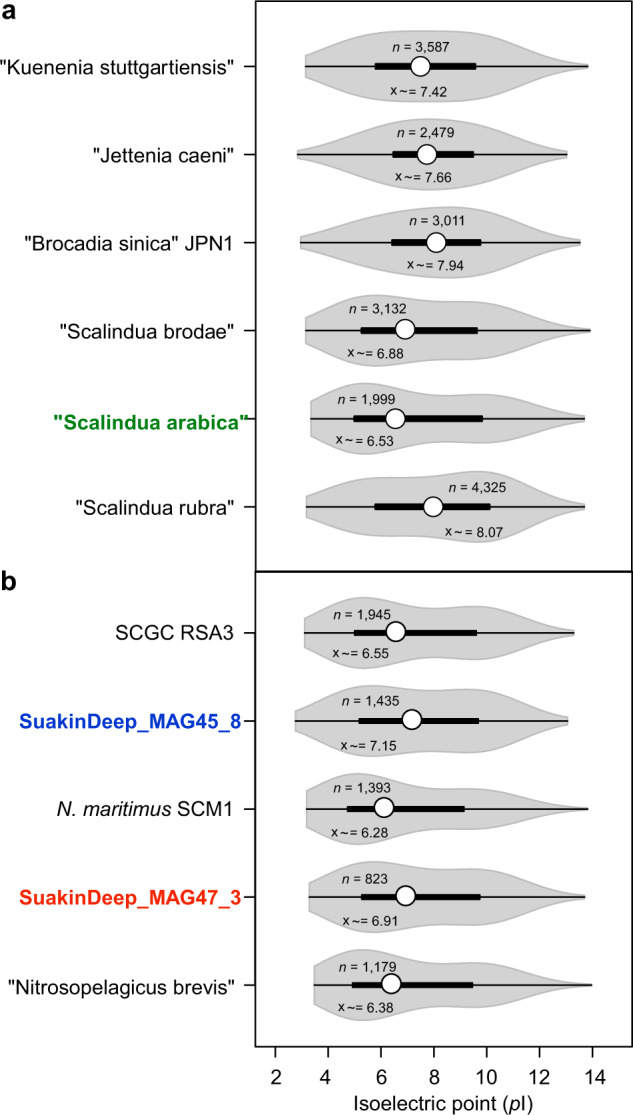
Genetic inventory of shared and conserved metabolic functions. **a**, **b** Violin plots showing the distribution in the isoelectric point (pI) of predicted cytoplasmatic proteins encoded in representative Anammox (**a**) and thaumarchaeal (**b**) genomes, including the reconstructed MAGs (shown in bold). Inside the violin plots, boxes (black bars) correspond to the first and third quartiles of the distribution, while the open circle corresponds to the median, and the whiskers extend to the extrema no further than 1.5 times the interquartile range. The total count of predicted proteins without transmembrane domains and their median pI (x̃) are shown.

The stratified distribution of AOA species and the anammox “*Ca*. Scalindua arabica” implies an inherent adaptation to the local challenging conditions along the BSI. Previous work showed that salinity is a significant factor confounding the distribution of nitrifying prokaryotes [21], with significant effects on the activity and growth of anammox bacteria [89]. To counteract the effects of salinity, microorganisms have developed two strategies, the “salt-in” and “salt-out”. The “salt-in” strategy consists of the accumulation of K^+^ in the cytoplasm and so requires the adaptation of the cellular machinery characterized by a charged cytoplasm and acid proteome [18, 21], whereas in the “salt-out” strategy, prokaryotes accumulate compatible solutes, such as ectoine, glycine betaine, and proline to balance the osmotic stress due to the high salt concentration [21]. Therefore, we examined whether the predicted proteomes of the three MAGs encode a charged cytoplasm characteristic of the “salt-in” strategy based on the pI metric (Fig. 6a, b). The analysis of cytoplasmatic proteins encompassing 70–81% of predicted proteome in each genome (2471–5458 protein-coding genes), revealed an acid shift of “*Ca*. Scalindua arabica” proteins (pI median of 6.53) than any other Anammox sequenced to date (Fig. 6a), including “*Ca*. Scalindua rubra” (pI median of 8.07) obtained from Discovery Deep [86]. This strongly suggested an adaptation to higher salinity, corroborating the high relative abundance of “*Ca*. Scalindua arabica” with increasing salinity of the BSI (Fig. 5c). In contrast, both the thaumarchaeal MAGs, *Nitrosopumilus* (SuakinDeep_MAG45_8) and *Nitrosopelagicus* (SuakinDeep_MAG47_3), possessed a more basic proteome (median pI of 7.34) compared to the other reference strains (median pI of 6.92–7.13; Fig. 6b), suggesting that osmoadaptation is likely afforded through the “salt-out” strategy. Indeed, a detailed profiling of metabolic pathways involved in the uptake and metabolism of compatible solutes indicates potential capacity to use osmolytes for the organisms studied here (Supplementary Table 7). For instance, anammox bacteria are predicted to encode the high-affinity betaine–choline–carnitine transport system and the ABC-type transporter for glycine betaine and proline (OpuAC). They also encode the putative machinery to synthesize proline and trehalose (Supplementary Table 7). In contrast, the thaumarchaeal genomes lacked many of these systems, with the exception of the ectoine/hydroxyectoine production pathway (EctABCD) that was present only in the *Nitrosopumilus* lineage (Supplementary Table 7). Together, these results suggest that scavenged (i.e., glycine betaine) or endogenously produced osmolytes (including ectoine) were likely solely used for osmoadaptation. Collectively, these results emphasize the significance of salinity in the ecology and biogeography of microorganisms involved in key nitrogen biogeochemical cycles of BSIs.

## Conclusions

In this study, we demonstrated the existence of a fine-scale stratification of the prokaryotic ensemble in the Suakin Deep and potential nitrogen processes that they catalyze, within a meter-scale pycnocline. The results also underscore the redoxcline as a pertinent driver of metabolic differentiation and community assembly in the halocline in line with other/previous works on oxygen minimum zones in the ocean. Furthermore, we reveal a potentially novel anammox bacterium, “*Ca*. Scalindua arabica” highly adapted to the salinity regimes in the investigated BSI. The study also reinforces previous results that showed the stratification of thaumarchaeal communities in DHABs. Additional high spatial resolution investigations in chemically diverse DHABs can expand this knowledge further to understand the ecology of these unique deep-sea habitats and their roles in the deep-sea realm, which can inform the nature of microbiome and enzymes that might shape analogous brines in extraterrestrial bodies [90, 91] or the processes that occur at the interface of oxygen minimum zone or oxygen-depleted ocean, such as the Baltic Sea [92].

## Supplementary information

Supplementary Figures

Supplementary Tables

Supplementary Table S7
