# Supplementary material for: Fine-scale metabolic discontinuity in a stratified prokaryote microbiome of a Red Sea deep halocline
Source: ISME J. 2021 Mar 1;15(8):2351–65. doi: 10.1038/s41396-021-00931-z (PMC8319295; doi:10.1038/s41396-021-00931-z)
Supplement: Supplementary file 1 — Supplementary Figures [file 41396_2021_931_MOESM1_ESM.pdf]

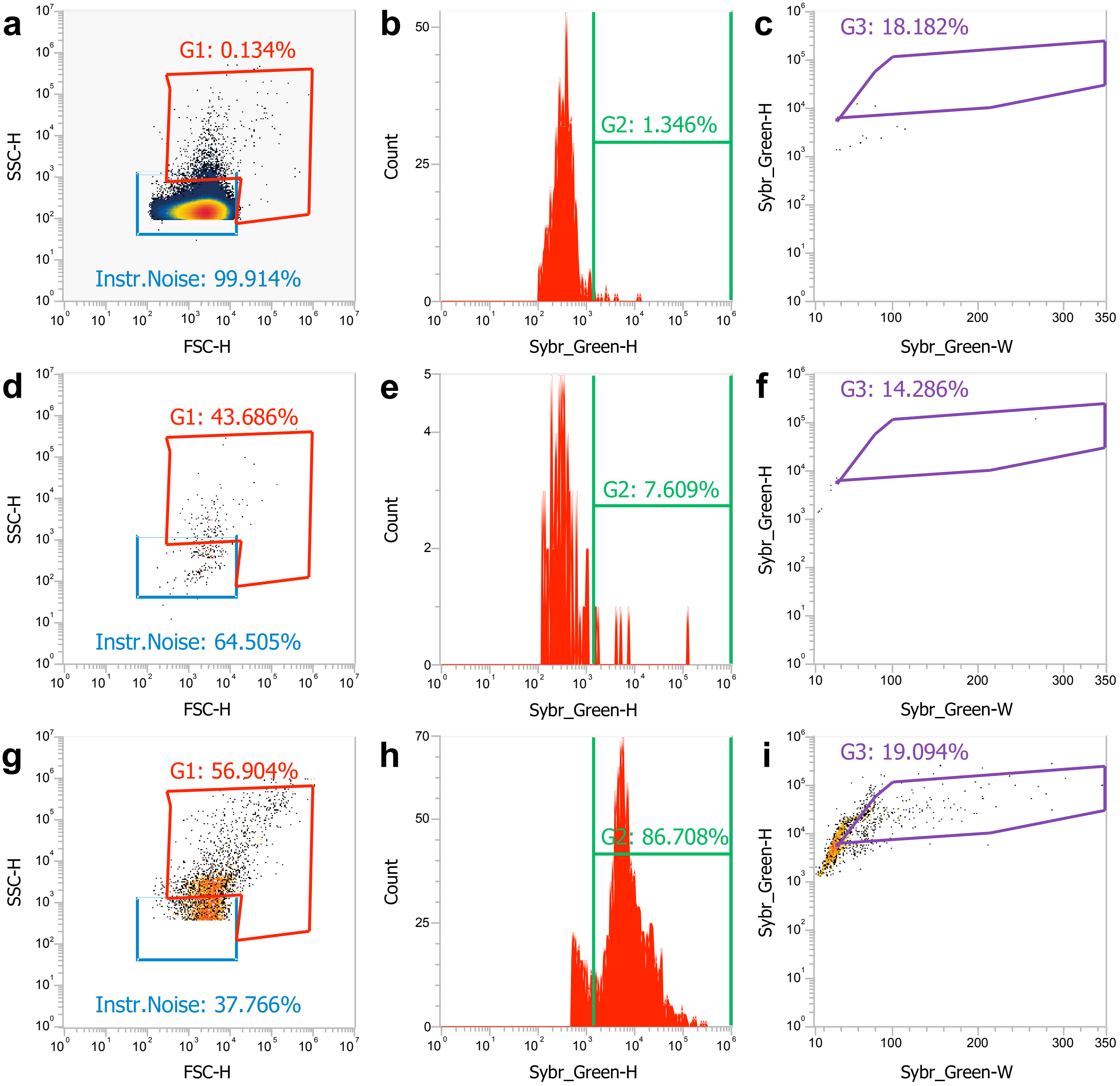

Fig. S1. Microbial cells count performed with Attune NxT. (**a, b, c**) Negative control without threshold setting. (**d, e, f**) Negative control with FSC 100, SSC 400 threshold setting. (**g, h, i**), Brine fraction sample. Negative control was filtered in 0.2 μm and stained with Sybr Green 10x.

Gate. Instr.Noise and G1 were set to exclude instrument noise events. G2 includes only events inside G1 and was set to exclude false fluorescent positive events. G3 was set to count cell doublets and include only event inside G2. Gating Strategy. Negative Control (**a**) was used as reference to set the gates and thresholds to avoid instrument noise on the different channels. Negative control (**e**) was used to set gate G2 to not take in account false fluorescent positive events. Events in gate G2 (**h**) were considered as total cells count for each fraction sample. Events in gate G3 (**i**) were considered cells aggregate (doublets) and were counted twice.

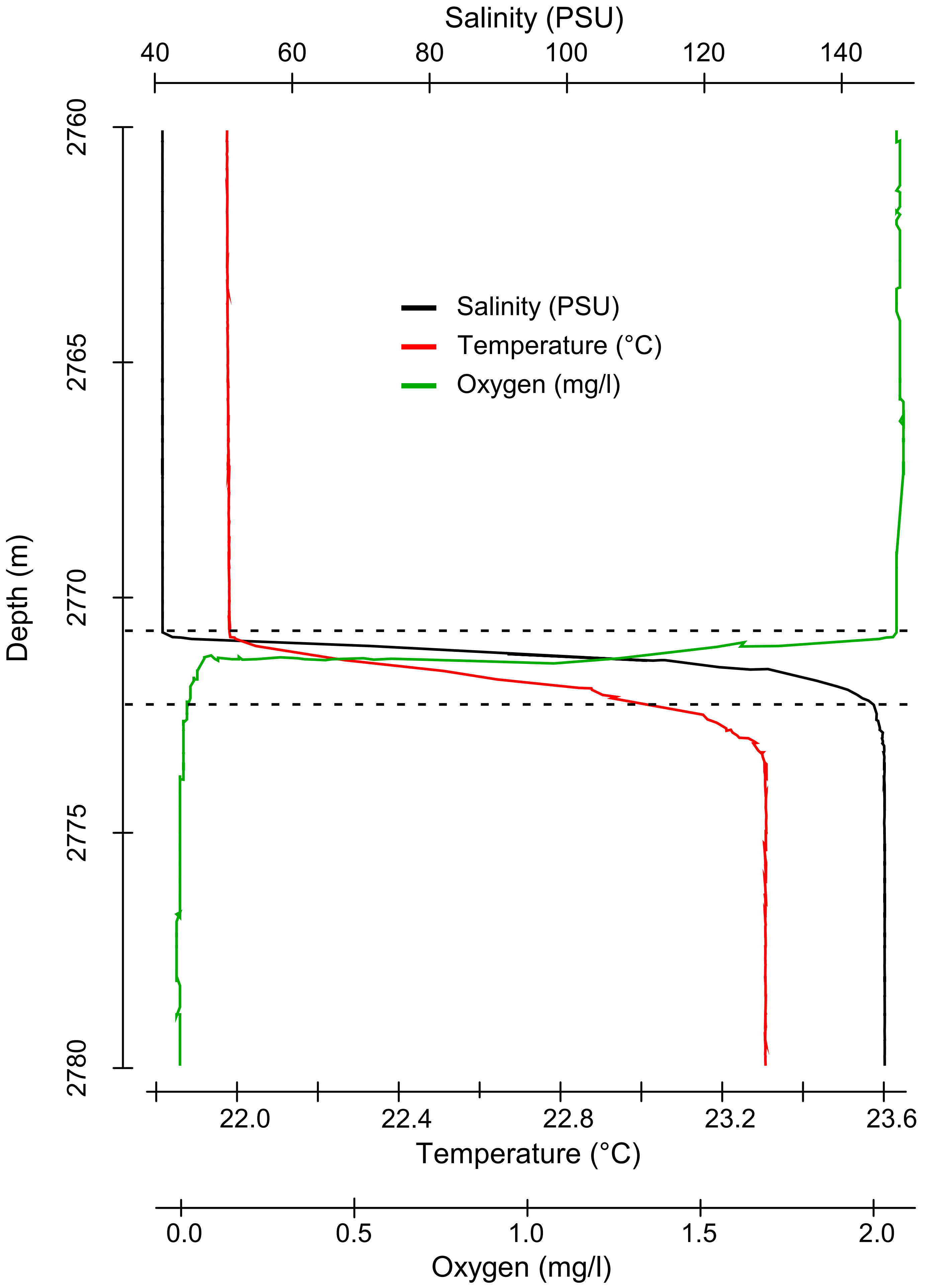

Fig. S2. Vertical profiles of physicochemical data (salinity, temperature and oxygen) along the Suakin brine in April 2016. The horizontal dashed lines denote the salinity transition zone.

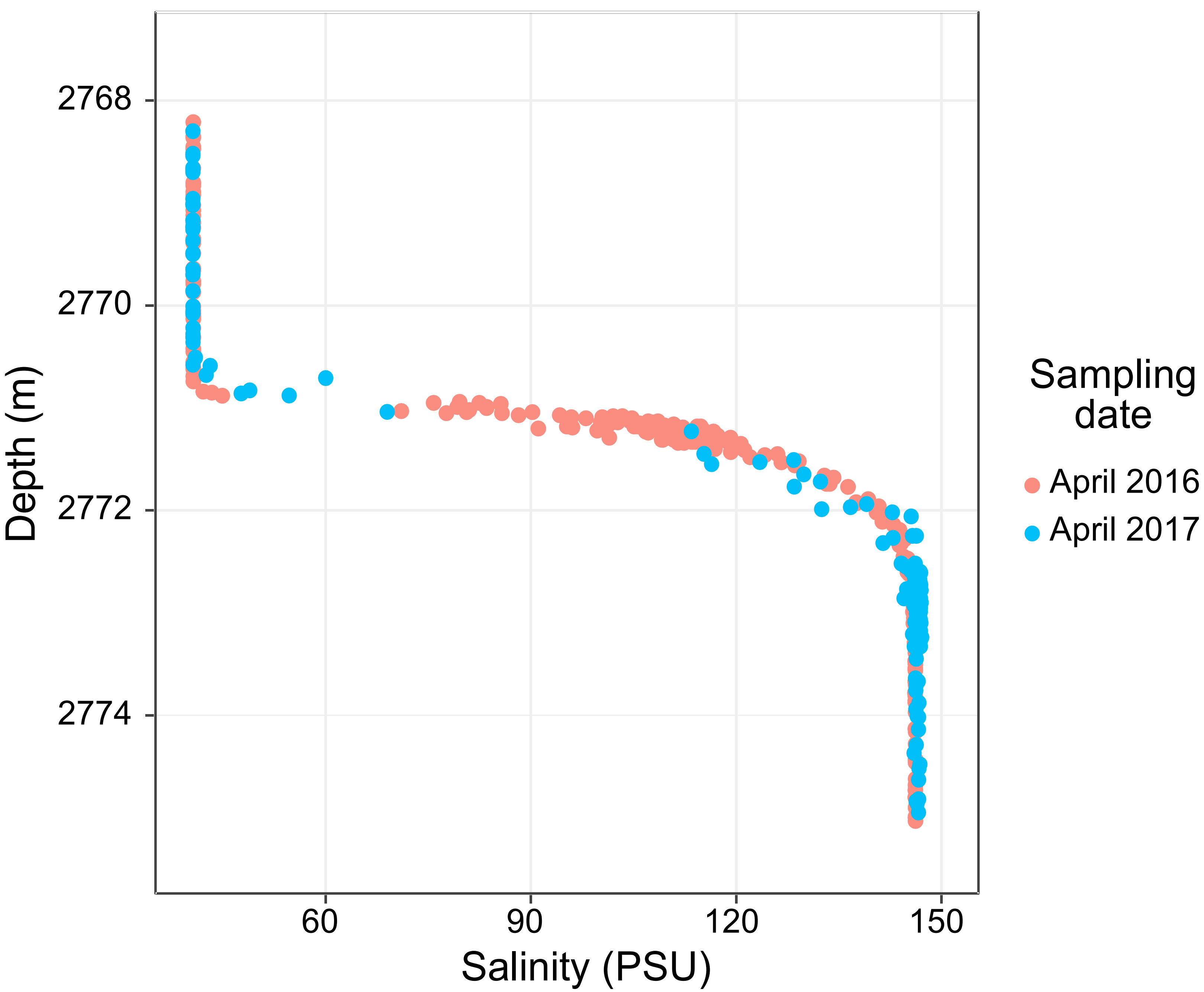

Fig. S3. Vertical profiles of salinity (CTD) obtained in April 2016 and 2017 along the Suakin brine.

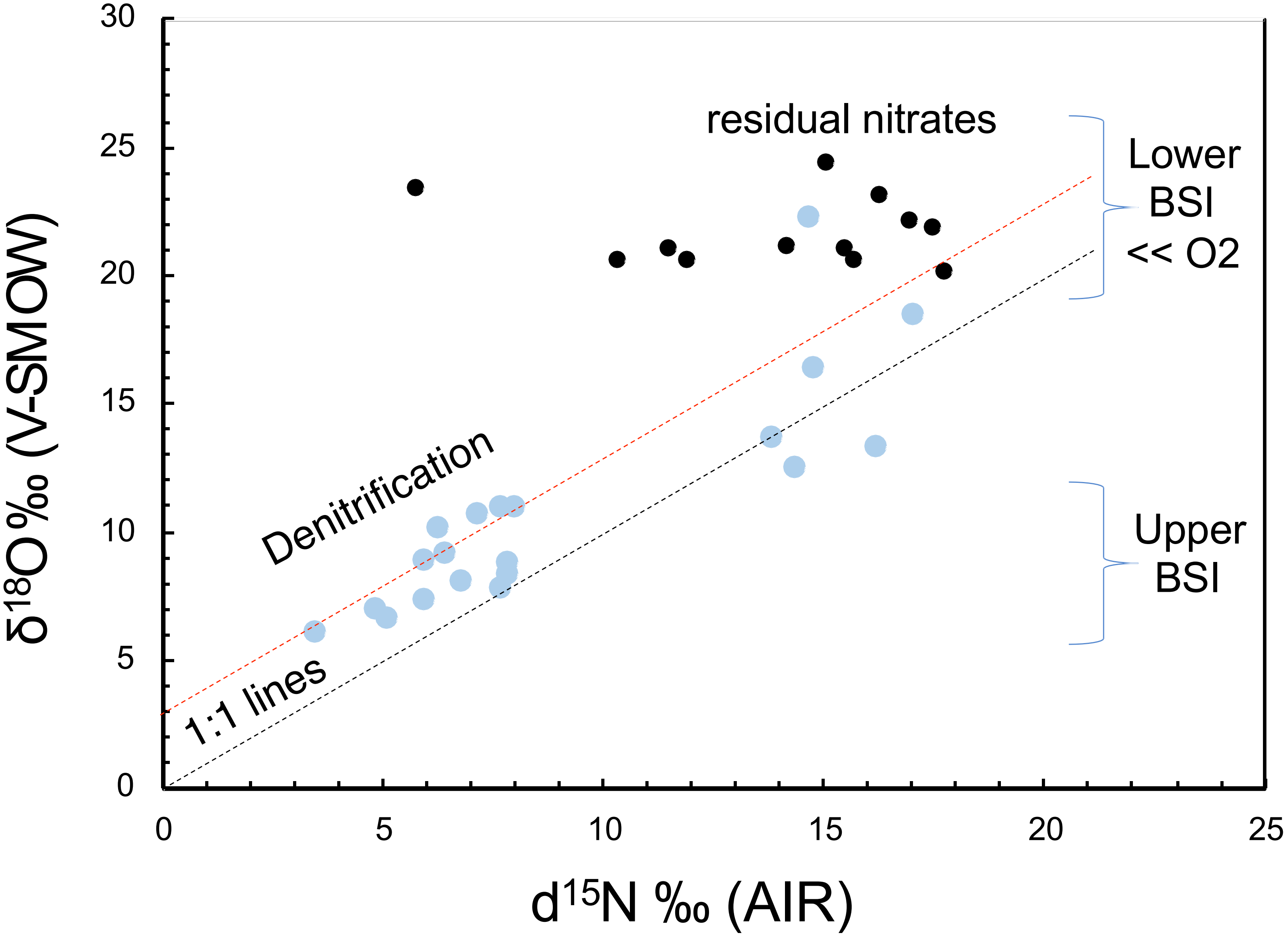

Fig. S4. Isotopic composition of nitrate. Blue circles represent samples with more than 3 μmol l^{-1} of NO_3^-. Black circles represent samples with less than 3 μmol l^{-1} of NO_3^- which are usually located in the Lower BSI (more anoxic). Lines 1: 1 with origin in zero and in the least positive samples (Δδ18O:Δδ15N trajectories) would represent the typical denitrification trend. Samples plotted at the left of the 1:1 line can be related with anammox processes

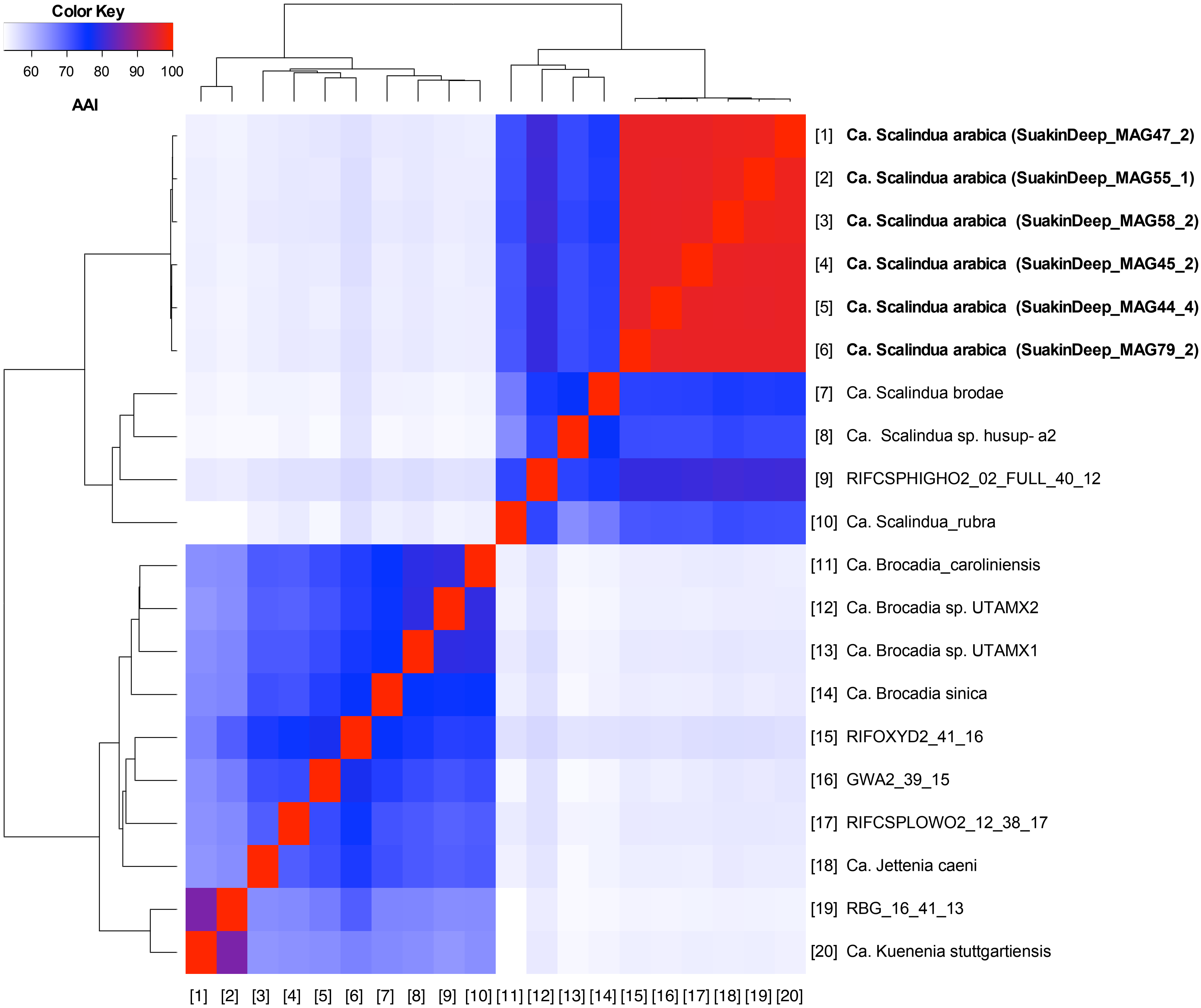

Fig. S5. Pairwise average amino acid identity (AAI) of reconstructed "*Candidatus* Scalindua arabica" MAGs and reference Anammox bacteria. The hierarchical clustering shows the separation of Scalindua species from other Anammox genera and the high AAI of reconstructed MAGs.

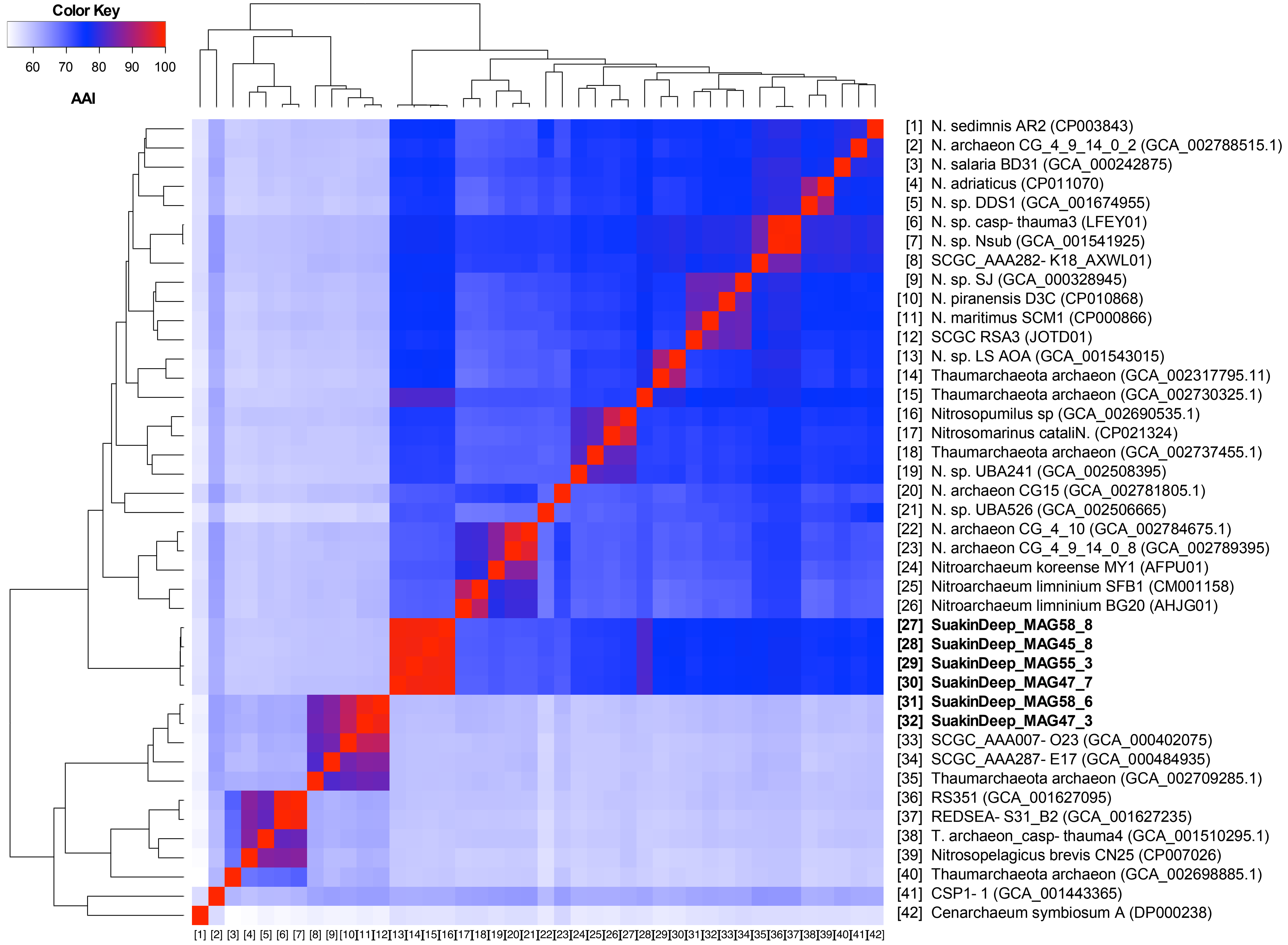

Fig. S6. Pairwise average amino acid identity (AAI) of reconstructed thaumarchaeal MAGs and closely related reference genomes.
