# Supplementary material for: Fine-scale metabolic discontinuity in a stratified prokaryote microbiome of a Red Sea deep halocline
Source: ISME J. 2021 Mar 1;15(8):2351–65. doi: 10.1038/s41396-021-00931-z (PMC8319295; doi:10.1038/s41396-021-00931-z)
Supplement: Supplementary file 2 — Supplementary Tables [file 41396_2021_931_MOESM2_ESM.pdf]

Table S1. General features and statistics of the metagenomes generated in this study.

Sample	Raw Reads	Sequence data (Gbp)	Qced Reads	Average Genome Size	Number of Contigs	N50 (bp)	Total contig length (bp)	ORFs Number	Accession data
BODY	22,403,713	3.4	14,983,272	3,514,526	6,391	568	19,282,125	22,399	SRR10589506
N4F2	26,735,052	4.0	21,837,300	4,320,848	13,644	1,385	26,467,659	33,911	SRR10589508
N4F3	24,140,106	3.6	21,663,244	3,414,299	8,968	883	17,696,763	23,379	SRR10589507
N4F4	37,242,987	5.6	34,840,106	3,358,096	10,177	768	33,378,403	38,125	SRR10589505
N4F5	46,421,983	7.0	44,660,036	3,668,819	13,473	856	38,510,036	45,426	SRR10589504
N4F7	38,739,953	5.8	21,820,406	2,490,042	11,557	987	25,919,841	33,840	SRR10589503
N5F5	28,578,140	4.3	26,401,618	2,686,281	8,604	432	19,672,565	26,165	SRR10589502
N5F8	37,439,268	5.7	26,000,116	2,485,489	16,403	1,098	30,482,097	42,259	SRR10589501
N7F1	33,099,138	5.0	29,791,104	3,850,190	11,603	986	28,123,877	33,710	SRR10589500
N7F6	16,536,434	2.5	14,637,412	3,608,057	6,115	609	13,971,221	17,280	SRR10589499
N7F9	30,360,456	4.6	29,371,272	3,545,303	12,264	1,057	32,732,078	38,859	SRR10589498

Table S2. List of KEGG ortholog (KO) Iabels interrogated in this study based on the annotated gene catalog.

Cycle	Metabolism	Step	KEGG	Gene
Carbon	Calvin cycle	(K00855+K01602)/2	K00855	phosphoribulokinase
			K01602	RuBisCO small chain
	Aerobic CH_4 oxidation		K08684	methane monooxygenase
	Aerobic respiration	(K02256 + K02262) / 2 + (K02274 + K02276) / 2	K02256	cytochrome c oxidase subunit I (coxI)
			K02262	cytochrome c oxidase subunit III (coxIII)
			K02274	cytochrome c oxidase subunit I (coxA)
			K02276	cytochrome c oxidase subunit III (coxC)
	reverse TCA cycle	(K00174 + K00175 + K00244 + K01648) / 4	K00174	2-oxoglutarate:ferredoxin oxidoreductase subunit alpha
			K00175	2-oxoglutarate:ferredoxin oxidoreductase subunit beta
			K00244	frdA; fumarate reductase flavoprotein subunit
			K01648	adenosinetriphosphate (ATP) citrate lyase
	Wood–Ljungdahl pathway	(K00194 + K00197) / 2	K00194	CO dehydrogenase subunit delta
			K00197	CO dehydrogenase subunit gamma
	CO oxidation	(K03518 + K03519 + K03520) / 3	K03518	CO dehydrogenase small subunit (coxS)
			K03519	cutM, coxM; carbon-monoxide dehydrogenase medium subunit
			K03520	cutL, coxL; carbon-monoxide dehydrogenase large subunit
	Methanogenesis	(K00400 + K00401) / 2	K00400	coenzyme M methyl reductase beta subunit (mcrB)
			K00401	methyl coenzyme M reductase system, component A2
Nitrogen	Ammonification	K05904 + K03385	K03385	formate-dependent nitrite reductase periplasmic cytochrome (nrfA)
			K05904	cytochrome c nitrite reductase (nrfA)
	Anammox	(K20933 + K20934) / 2	K20933	hydrazine synthase subunit
			K20934	hydrazine synthase subunit
	Denitrification	(K02305 + K04561 + K00376) / 3	K00376	nitrous oxide reductase (nosZ)
			K02305	nitric-oxide reductase (norC)
			K04561	nitric-oxide reductase (norB)
	Nitrite oxidation		K00370	nitrite oxidoreductase (nxrA)
	Nitrate reduction	K02567 + K00370	K02567	periplasmic nitrate reductase (napA)
			K00370	nitrate reductase alpha (narG)
	Nitrification	(K10944 + K10945 + K10946) / 3	K10944	ammonia monooxygenase subunit A (amoA)
			K10945	ammonia monooxygenase subunit B (amoB)
			K10946	ammonia monooxygenase subunit C (amoC)
	Nitrogen assimilation	(K00360 + K00367 + K01915 + K00265 + K00284) / 3	K00265	glutamate synthase (NADPH/NADH) large chain (gltB)
			K00284	glutamate synthase (ferredoxin-dependent) (gltS)
			K00360	assimilatory nitrate reductase
			K00367	assimilatory nitrate reductase
			K01915	glutamine synthetase (glnA)
	Nitrogen Fixation	(K00531 + K02586 + K02588 + K02591)/4	K00531	nitrogenase
			K02586	nitrogenase molybdenum-iron protein alpha chain (nifD)
			K02588	nitrogenase iron protein (nifH)
			K02591	nitrogenase molybdenum-iron protein beta chain (nifK)
Sulfur	Assimilatory sulfate reduction	(K00860 + K00956 + K00957) / 3	K00860	adenylylsulfate kinase (cysC)
			K00956	sulfate adenylyltransferase subunit 1 (cysN)
			K00957	sulfate adenylyltransferase subunit 2 (cysD)
	Sulfide oxidation		K17218	sulfide:quinone oxidoreductase
	Dissimilatory sulfate reduction		K11180	sulfite reductase (dsrA)
	Sulfur Mineralization	K00456 + K01011	K00456	cysteine dioxygenase
			K01011	3-mercaptopyruvate sulfurtransferase
	Polysulfide reduction		K08352	polysulfide reductase chain A (psrA)

Table S3. Spearman correlation between all the environmental variables measured along the BSI in 2016 (**a**) and 2017 (**b**). The values in bold characterize significant correlations ($P < 0.01$).

a

	**Depth (m)**	**Salinity (PSU)**	Al (nmol l^{-1})	Ca (mmol l^{-1})	Cd (nmol l^{-1})	Cl (mmol l^{-1})	Co (nmol l^{-1})	Cu (nmol l^{-1})	DOC (µmol l^{-1})	Fe (nmol l^{-1})	Mg (mmol l^{-1})	Mn (nmol l^{-1})	Na (mmol l^{-1})
**Depth**	1												
**Salinity**	**0.99**	1											
Al	-0.28	-0.27	1										
Ca	**0.99**	**1**	-0.27	1									
Cd	**0.58**	**0.56**	-0.43	**0.56**	1								
Cl	**0.99**	**1**	-0.28	**1**	**0.56**	1							
Co	**0.80**	**0.82**	-0.45	**0.82**	**0.72**	**0.82**	1						
Cu	0	0	0.09	0	-0.06	0	0.1	1					
DOC	**-0.80**	**-0.74**	0.3	**-0.74**	**-0.65**	**-0.74**	**-0.72**	0.09	1				
Fe	-0.06	-0.07	0.06	-0.07	0.06	-0.07	0.01	0.49	0.06	1			
Mg	**0.99**	**1**	-0.27	**1**	**0.56**	**1**	**0.82**	0	**-0.74**	-0.07	1		
Mn	**0.77**	**0.77**	**-0.52**	**0.77**	**0.74**	**0.78**	**0.97**	0.18	-0.61	0.08	**0.77**	1	
Na	**0.99**	**1**	-0.28	**1**	**0.56**	**1**	**0.82**	0	**-0.74**	-0.07	**1**	**0.78**	1
NH_4^+	-0.2	-0.21	-0.23	-0.21	0.07	-0.21	0	0.07	-0.15	0.1	-0.21	0.02	-0.21
Ni	**0.64**	**0.63**	**-0.51**	**0.63**	**0.75**	**0.63**	**0.86**	0.16	**-0.71**	0.06	**0.63**	**0.84**	**0.63**
NO_2^-	**0.66**	**0.66**	-0.29	**0.66**	0.3	**0.66**	0.45	0.23	-0.1	0.15	**0.66**	**0.50**	**0.66**
NO_3^-	**-0.80**	**-0.81**	0.24	**-0.81**	**-0.51**	**-0.81**	**-0.69**	-0.03	**0.66**	0.03	**-0.81**	**-0.61**	**-0.81**
Pb	**0.62**	**0.62**	-0.08	**0.62**	0.21	**0.62**	**0.53**	0.15	-0.33	0.09	**0.62**	**0.51**	**0.62**
pH	**-0.65**	**-0.66**	0.42	**-0.66**	**-0.66**	**-0.66**	**-0.67**	-0.13	0.61	-0.26	**-0.66**	**-0.65**	**-0.66**
PO_4^{-3}	-0.15	-0.15	0.19	-0.15	-0.43	-0.14	-0.17	0.18	0.55	0.16	-0.15	-0.09	-0.14
SiO_2	**0.90**	**0.89**	-0.23	**0.89**	**0.55**	**0.89**	**0.76**	0.07	**-0.83**	0.02	**0.89**	**0.72**	**0.89**
SO_4^{-2}	**0.99**	**1**	-0.27	**1**	**0.56**	**1**	**0.82**	0	**-0.74**	-0.07	**1**	**0.77**	**1**
Sr	**0.99**	**1**	-0.28	**1**	**0.56**	**1**	**0.82**	0	**-0.74**	-0.07	**1**	**0.78**	**1**
TDN	**0.97**	**0.99**	-0.12	**0.99**	0.39	**0.99**	**0.72**	0.11	**-0.69**	0.08	**0.99**	0.62	**0.99**
Urea	**0.84**	**0.85**	-0.31	**0.85**	0.49	**0.85**	**0.71**	-0.09	**-0.71**	-0.1	**0.85**	**0.65**	**0.85**
Zn	**0.62**	**0.62**	**-0.55**	**0.62**	**0.72**	**0.63**	**0.89**	0.27	-0.51	0.19	**0.62**	**0.91**	**0.63**

	NH_4^+ (µmol l^{-1})	Ni (nmol l^{-1})	NO_2^- (µmol l^{-1})	NO_3^- (µmol l^{-1})	Pb (pmol l^{-1})	pH	PO (µmol l^{-1})	SiO_2 (µmol l^{-1})	SO_4^{-2} (mmol l^{-1})	Sr (µmol l^{-1})	TDN (µmol l^{-1})	Urea (nmol l^{-1})	Zn (nmol l^{-1})
NH_4^+	1												
Ni	0.22	1											
NO_2^-	-0.12	0.29	1										
NO_3^-	0.12	**-0.57**	**-0.59**	1									
Pb	-0.17	0.3	**0.56**	-0.44	1								
pH	-0.23	**-0.69**	-0.49	**0.78**	-0.26	1							
PO_4^{-3}	-0.39	-0.4	0.06	0.43	0.2	**0.57**	1						
SiO_2	-0.16	**0.67**	0.46	**-0.61**	0.48	**-0.55**	-0.08	1					
SO_4^{-2}	-0.21	**0.63**	**0.66**	**-0.81**	**0.62**	**-0.66**	-0.15	**0.89**	**1**				
Sr	-0.21	**0.63**	**0.66**	**-0.81**	**0.62**	**-0.66**	-0.14	**0.89**	1				
TDN	0	0.61	0.61	**-0.86**	**0.67**	**-0.72**	-0.28	**0.89**	**0.99**	**0.99**	1		
Urea	-0.07	**0.54**	**0.65**	**-0.89**	0.43	**-0.73**	-0.32	**0.63**	**0.85**	**0.85**	**0.97**	1	
Zn	0.19	**0.92**	0.4	**-0.54**	0.39	**-0.72**	-0.26	**0.63**	**0.63**	**0.62**	0.5	**0.50**	1

b

	**Depth (m)**	δ15N (AIR)	δ18O (SMOW)
**Depth**	1		
δ15N	**0.84**	1	
δ18O	**0.71**	**0.70**	1

Table S4. General statistics of the high-quality MAGs (≥ 75% complete; ≤ 5% contamination) along with their putative taxonomy.

MAG	Completeness (%)	Contamination (%)	Strain heterogeneity (%)	Length (bp)	N50 (bp)	Estimated Length (bp)
SuakinDeep_MAG42_1	95.6	0	0	2,736,927	16,519	2,862,894
SuakinDeep_MAG44_1	87.51	0	0	2,474,173	7,736	2,827,303
SuakinDeep_MAG45_3	96.7	0	0	2,845,984	24,817	2,943,107
SuakinDeep_MAG71_1	91.76	1.1	0	2,671,604	11,303	2,911,513
SuakinDeep_MAGBody_1	92.31	1.1	0	2,844,301	15,656	3,081,249
SuakinDeep_MAG45_4	77.35	1.28	0	1,486,851	66,989	1,922,238
SuakinDeep_MAG47_1	91.88	1.28	0	1,863,001	34,783	2,027,646
SuakinDeep_MAG58_3	90.1	1.28	0	1,867,866	42,060	2,073,103
SuakinDeep_MAG43_1	87.27	2	0	2,710,951	27,648	3,106,395
SuakinDeep_MAG44_2	86.82	2.91	0	2,954,652	39,622	3,403,193
SuakinDeep_MAG45_1	75.23	2.02	0	3,832,981	10,981	5,095,017
SuakinDeep_MAG79_3	75	1.82	0	2,032,210	24,804	2,709,613
SuakinDeep_MAG45_8	94.17	0.97	0	1,374,776	31,909	1,459,887
SuakinDeep_MAG47_3	78.42	1.94	100	824,129	12,505	1,050,917
SuakinDeep_MAG47_7	86.08	1.05	50	1,151,007	19,711	1,337,136
SuakinDeep_MAG55_3	89.97	0.97	0	1,418,335	42,150	1,576,453
SuakinDeep_MAG58_8	87.06	0.97	0	1,264,290	18,547	1,452,205
SuakinDeep_MAG42_3	79.52	1.31	0	1,123,369	77,313	1,412,687
SuakinDeep_MAG71_5	82.79	1.31	0	1,171,120	72,822	1,414,567
SuakinDeep_MAGBody_3	82.22	1.96	33.33	1,094,012	47,469	1,330,591
SuakinDeep_MAGBody_4	81.32	1.1	0	1,935,300	12,005	2,379,857
SuakinDeep_MAG79_5	89.25	0	0	1,167,524	33,157	1,308,150
SuakinDeep_MAG44_4	89.49	2.84	33.33	3,007,171	41,361	3,360,343
SuakinDeep_MAG45_2	91.76	2.58	60	2,910,846	36,298	3,172,238
SuakinDeep_MAG47_2	79.26	1.34	66.67	2,109,492	29,437	2,661,484
SuakinDeep_MAG55_1	92.9	2.27	0	2,693,846	34,459	2,899,727
SuakinDeep_MAG58_2	82.67	2.94	50	2,329,318	14,823	2,817,610
SuakinDeep_MAG79_2	81.82	2.27	50	2,736,049	29,603	3,343,986
SuakinDeep_MAG44_5	89.48	0	0	2,165,280	22,309	2,419,848
SuakinDeep_MAG79_4	78.66	0.04	0	1,934,733	14,224	2,459,615
SuakinDeep_MAG58_1	88.7	0.05	100	3,038,269	68,596	3,425,331

MAG	Domain	Phylum	Class	Order	Family	Genus	Species
SuakinDeep_MAG42_1	Bacteria	AABM5-125-24	AABM5-125-24				
SuakinDeep_MAG44_1	Bacteria	AABM5-125-24	AABM5-125-24				
SuakinDeep_MAG45_3	Bacteria	AABM5-125-24	AABM5-125-24				
SuakinDeep_MAG71_1	Bacteria	AABM5-125-24	AABM5-125-24				
SuakinDeep_MAGBody_1	Bacteria	AABM5-125-24	AABM5-125-24				
SuakinDeep_MAG45_4	Bacteria	Actinobacteriota	Acidimicrobiia	Microtrichales	MedAcidi-G1	UBA3125	GCA_002687745.1
SuakinDeep_MAG47_1	Bacteria	Actinobacteriota	Acidimicrobiia	Microtrichales	MedAcidi-G1	UBA3125	GCA_002687745.1
SuakinDeep_MAG58_3	Bacteria	Actinobacteriota	Acidimicrobiia	Microtrichales	MedAcidi-G1	UBA3125	GCA_002687745.1
SuakinDeep_MAG43_1	Bacteria	Chloroflexota	Anaerolineae	Anaerolineales	UBA11858		
SuakinDeep_MAG44_2	Bacteria	Chloroflexota	Anaerolineae	Anaerolineales	UBA11858		
SuakinDeep_MAG45_1	Bacteria	Chloroflexota	Anaerolineae	UBA7937			
SuakinDeep_MAG79_3	Bacteria	Chloroflexota	Anaerolineae	Anaerolineales	UBA11858		
SuakinDeep_MAG45_8	Archaea	Crenarchaeota	Nitrososphaeria	Nitrososphaerales	Nitrosopumilaceae	Nitrosopumilus	
SuakinDeep_MAG47_3	Archaea	Crenarchaeota	Nitrososphaeria	Nitrososphaerales	Nitrosopumilaceae	Nitrosopelagicus	
SuakinDeep_MAG47_7	Archaea	Crenarchaeota	Nitrososphaeria	Nitrososphaerales	Nitrosopumilaceae	Nitrosopumilus	
SuakinDeep_MAG55_3	Archaea	Crenarchaeota	Nitrososphaeria	Nitrososphaerales	Nitrosopumilaceae	Nitrosopumilus	
SuakinDeep_MAG58_8	Archaea	Crenarchaeota	Nitrososphaeria	Nitrososphaerales	Nitrosopumilaceae	Nitrosopumilus	
SuakinDeep_MAG42_3	Archaea	Halobacterota	Methanonatronarchaeia				
SuakinDeep_MAG71_5	Archaea	Halobacterota	Methanonatronarchaeia				
SuakinDeep_MAGBody_3	Archaea	Halobacterota	Methanonatronarchaeia				
SuakinDeep_MAGBody_4	Bacteria	Marinisomatota					
SuakinDeep_MAG79_5	Archaea	Nanoarchaeota	Woesearchaeia	GW2011-AR15	GW2011-AR15		
SuakinDeep_MAG44_4	Bacteria	Planctomycetota	Brocadiae	Brocadiales	Scalinduaceae	Scalindua	
SuakinDeep_MAG45_2	Bacteria	Planctomycetota	Brocadiae	Brocadiales	Scalinduaceae	Scalindua	

MAG	Domain	Phylum	Class	Order	Family	Genus	Species
SuakinDeep_MAG47_2	Bacteria	Planctomycetota	Brocadiae	Brocadiales	Scalinduaceae	Scalindua	
SuakinDeep_MAG55_1	Bacteria	Planctomycetota	Brocadiae	Brocadiales	Scalinduaceae	Scalindua	
SuakinDeep_MAG58_2	Bacteria	Planctomycetota	Brocadiae	Brocadiales	Scalinduaceae	Scalindua	
SuakinDeep_MAG79_2	Bacteria	Planctomycetota	Brocadiae	Brocadiales	Scalinduaceae	Scalindua	
SuakinDeep_MAG44_5	Bacteria	Proteobacteria	Gammaproteobacteria	UBA10353			
SuakinDeep_MAG79_4	Bacteria	Proteobacteria	Gammaproteobacteria	UBA10353			
SuakinDeep_MAG58_1	Bacteria	SAR324	SAR324	SAR324	NAC60-12	Arctic96AD-7	Arctic96AD-7sp3

Table S5. Geochemical data of the BSI of Suakin Deep obtained in 2016 (**a**) and 2017 (**b**).

a

**Fraction**	**Depth (m)**	**Salinity (PSU)**	Al (nmol l^{-1})	Ca (mmol l^{-1})	Cd (nmol l^{-1})	Cl (mmol l^{-1})	Co (nmol l^{-1})	Cu (nmol l^{-1})	DOC (µmol l^{-1})	Fe (nmol l^{-1})	Mg (mmol l^{-1})	Mn (nmol l^{-1})	Na (mmol l^{-1})	NH_4^+ (µmol l^{-1})
**N5F8**	**2,770.61**	**41.01**	103.3	13.3	0.99	708.5	1.89	0.15	47.17	3.75	68.6	877.26	608.7	1.3
**N4F7**	**2,770.84**	**41.71**	83.61	13.6	3.47	720.7	1.75	2.96	47.47	9.94	69.7	5,087.03	619.2	0.4
**N5F5**	**2,770.89**	**47.95**	92.18	15.6	2.42	828.4	3.01	<0.3	48.54	2.9	80.2	21,901.42	711.7	5.53
**N4F5**	**2,771.02**	**70.93**	8.28	23.1	2.19	1,225.4	5.78	<0.3	43.38	7.79	118.6	138,188.06	1,052.8	11.14
**N4F4**	**2,771.11**	**96.85**	65.68	31.5	5.95	1,673.2	11.4	4.72	46.28	11.5	161.9	305,489.13	1,437.5	5.19
**N4F3**	**2,771.21**	**117.58**	56.18	38.2	3.9	2,031.4	15.19	1.42	43.45	17.48	196.6	446,994.9	1,745.3	2.07
**N7F9**	**2,771.28**	**110.72**	6.14	36	9.72	1,913	12.66	<0.3	36.65	5.21	185.1	385,726.09	1,643.5	6.04
**N4F2**	**2,771.3**	**124.78**	82.98	40.6	2.92	2,155.9	9.51	3.88		15.69	208.7	270,663.94	1,852.2	9.17
**N7F6**	**2,771.56**	**129.33**	28.15	42.1	6.14	2,234.4	16.88	<0.3	40.69	<5	216.3	492,845.37	1,919.7	1.77
**N7F1**	**2,772.02**	**139.33**	172.87	45.3	<0.5	2,407.2	2.6	<0.3	43.35	<5	233.0	1,663.64	2,068.1	0.84
**BODY**	**2,772.5**	**149.9**	125.23	45.4	2.71	2,413.1	12.25	0.99	43.44	17.37	233.5	484,399.2	2,073.2	0.32

**Fraction**	**Depth (m)**	**Salinity (PSU)**	Ni (nmol l^{-1})	NO_2^- (µmol l^{-1})	NO_3^- (µmol l^{-1})	Pb (pmol l^{-1})	pH	PO_4^{-3} (µmol l^{-1})	SiO_2 (µmol l^{-1})	SO_4^{-2} (mmol l^{-1})	Sr (µmol l^{-1})	TDN (µmol l^{-1})	Urea (nmol l^{-1})	Zn (nmol l^{-1})
**N5F8**	**2,770.61**	**41.01**	10.39	0.06	4.67	<10	7.9	0.32	5.51	36.6	117.5	13.04	628.1	18.99
**N4F7**	**2,770.84**	**41.71**	9.35	0.1	14.07	<10	7.89	0.47	16.53	37.3	119.6	16.91	245.6	74.23
**N5F5**	**2,770.89**	**47.95**	21.71	0.09	8.03	93.66	7.73	0.41	12.97	42.8	137.4	61.27	372.4	138.94
**N4F5**	**2,771.02**	**70.93**	25.72	0.06	9.86	<10	7.56	0.3	48.34	63.4	203.3	113.71	485	343.33
**N4F4**	**2,771.11**	**96.85**	52.85	0.07	5.02	<10	7.27	0.26	64.52	86.5	277.6	228.71	472.5	880.86
**N4F3**	**2,771.21**	**117.58**	59.68	0.09	1.17	<10	7.14	0.31	89.68	105.1	337	262.93	665.1	1,015.64
**N7F9**	**2,771.28**	**110.72**	62.59	0.08	2.35	18.65	7.22	0.27	78.41	98.9	317.3	236.93	581.4	989.64
**N4F2**	**2,771.3**	**124.78**	34.82	0.18	0.42	17.02	7.15	0.3	47.38	111.5	357.7		1,729.4	507.1
**N7F6**	**2,771.56**	**129.33**	50.73	0.15	0.09	33.17	7.18	0.24	38.48	115.6	370.7	288	1,017.7	968.79
**N7F1**	**2,772.02**	**139.33**	11.11	0.14	0.49	74.6	7.39	0.37	113.99	124.5	399.3	365.93	802.3	28.22
**BODY**	**2,772.5**	**149.9**	32.87	0.24	0	656.37	7.59	0.88	82.4	124.8	400.3	504.21	5,302.3	324.7

b

**Depth (m)**	δ15N NO_3^-	δ18O NO_3^-	**Depth (m)**	δ15N NO_3^-	δ18O NO_3^-
2770.33	8.06	6.79	2771.65	8.81	7.83
2770.52	7.32	5.91	2771.72	16.34	14.75
2770.7	7.75	7.69	2771.72	20.52	11.92
2770.89	10.99	7.99	2771.8	18.45	17.01
2770.91	6.58	5.1	2771.82	21.07	14.17
2771	7	4.83	2771.89	22.23	14.65
2771.07	10.91	7.68	2771.91	23.35	5.79
2771.1	6.11	3.48	2771.98	21.84	17.49
2771.19	10.18	6.27	2772	24.3	15.07
2771.28	8.84	5.92	2772.06	20.57	15.69
2771.37	9.13	6.41	2772.09	23.1	16.28
2771.47	10.71	7.15	2772.17	21.03	15.48
2771.52	12.44	14.34	2772.19	22.11	16.99
2771.56	8.34	7.82	2772.26	20.09	17.77
2771.61	13.64	13.83	2772.28	20.55	10.33
2771.63	13.31	16.17	2772.37	21.03	11.51

Table S6. Results of test for significance of environmental variables for the CCA.

	Df	AIC	F	Pr(>F)	Significance Codes	Significant
NH_4^+ (µmol l^{-1})	1	96.53	2.95	0.005	**	Yes
Salinity (PSU)	1	99.13	5.87	0.005	**	Yes
pH	1	94.39	1.94	0.055	.	No
PO_4^{-3} (mmol l^{-1})	1	94.39	1.94	0.06	.	No
Cd (nmol l^{-1})	1	94.77	1.63	0.09	.	No
Al (nmol l^{-1})	1	95.23	1.28	0.25		No
Fe (nmol l^{-1})	1	95.99	0.72	0.76		No
Cu (nmol l^{-1})	1	96.34	0.48	0.91		No
